# CircDIDO1 inhibits gastric cancer progression by encoding a novel DIDO1-529aa protein and regulating PRDX2 protein stability

**DOI:** 10.1186/s12943-021-01390-y

**Published:** 2021-08-12

**Authors:** Yu Zhang, Jiajia Jiang, Jiayin Zhang, Han Shen, Maoye Wang, Zhen Guo, Xueyan Zang, Hui Shi, Jiayan Gao, Hui Cai, Xinjian Fang, Hui Qian, Wenrong Xu, Xu Zhang

**Affiliations:** 1 Aoyang Cancer Institute, Affiliated Aoyang Hospital of Jiangsu University, Zhangjiagang, 215600 Jiangsu China; 2 Jiangsu Key Laboratory of Medical Science and Laboratory Medicine, School of Medicine, Jiangsu University, Zhenjiang, 212013 Jiangsu China; 3 Department of Laboratory Medicine, Nanjing Drum Tower Hospital, Nanjing University Medical School, Nanjing, 210009 Jiangsu China; 4 Department of Oncology, Lianyungang Hospital Affiliated To Jiangsu University, Lianyungang, 222000 Jiangsu China; 5 Key Laboratory of Molecular Diagnostics and Precision Medicine for Surgical Oncology in Gansu Province, Gansu Hospital of Jiangsu University, Lanzhou, 730000 Gansu China

**Keywords:** CircRNA, DIDO1, Gastric cancer, Biomarker, Target

## Abstract

**Background:**

Circular RNAs (circRNAs) play important roles in cancer development and progression. The purpose of this study is to identify aberrantly expressed circRNAs in gastric cancer (GC), unravel their roles in GC progression, and provide new targets for GC diagnosis and therapy.

**Methods:**

Bioinformatic analyses were performed to identify the aberrantly expression of hsa_circ_0061137 (termed as circDIDO1) in GC. Gain- and loss-of-function studies were performed to examine the biological roles of circDIDO1 in GC progression. Tagged RNA affinity purification, mass spectrometry, immunofluorescence, co-immunoprecipitation, and Western blot were used to identify circRNA-interacting and circRNA-encoded proteins. RNA sequencing, qRT-PCR, and Western blot were performed to analyze circRNA-regulated downstream target genes and signaling pathways. Mouse tumor models were used to analyze the effects of circDIDO1 on GC growth and metastasis.

**Results:**

CircDIDO1 was transcribed from human *DIDO1* (death-inducer obliterator 1) gene and formed by back-splicing of exons 2–6 of the linear transcript. circDIDO1 was down-regulated in GC tissues and its low levels were associated with larger tumor size, distal metastasis, and poor prognosis. CircDIDO1 overexpression inhibited while knockdown promoted GC cell proliferation, migration and invasion*.* CircDIDO1 overexpression suppressed GC growth and metastasis in mouse tumor models. Mechanistically, circDIDO1 encoded a novel 529aa protein that directly interacted with poly ADP-ribose polymerase 1 (PARP1) and inhibited its activity. CircDIDO1 also specifically bound to peroxiredoxin 2 (PRDX2) and promoted RBX1-mediated ubiquitination and degradation of PRDX2, which led to the inactivation of its downstream signaling pathways.

**Conclusions:**

CircDIDO1 is a new circRNA that has tumor suppressor function in GC and it may serve as a potential prognostic biomarker and therapeutic target for GC.

**Supplementary Information:**

The online version contains supplementary material available at 10.1186/s12943-021-01390-y.

## Background

Gastric cancer (GC) is one of the most common cancers and the fourth leading cause of cancer-related death [1]. Due to the lack of early detection, most GC patients are diagnosed at advanced stages and the prognosis is still unsatisfactory. In the past decades, despite tremendous efforts have been made to improve the diagnosis and treatment of GC, the incidence and mortality of GC remains high [2].

Circular RNAs (circRNAs), a group of transcripts characterized by covalently closed structure, are generated from exons or introns of their parental genes [3]. Although the function of circRNAs is not fully understood, recent studies have shown that circRNAs participate in many physiological and pathological processes by regulating epigenetic modification, gene transcription, alternative splicing, RNA stability, and protein translation [4]. Increasing evidence suggest that circRNAs are involved in cancer development and progression through distinct mechanisms [5–8]. For example, circHIPK3 functions as an autophagy regulator by competitively binding to miR-124 to facilitate lung cancer progression [5]. Circ-Foxo3 suppresses cell cycle progression by acting as a scaffold to promote p21/CDK2 interaction [6]. In addition, circRNAs have been found to encode proteins, such as circFBXW7, circPINT and circPPP1R12A, which further expands our understanding of their roles in cancers [9–11]. Moreover, circRNAs are found stably present in the circulation of cancer patients, making them candidate biomarkers for cancer diagnosis.

A growing number of circRNAs have been reported in GC and show promising diagnostic and prognostic values [12]. The deregulated circRNAs participate in many biological processes of GC by acting as miRNA sponges, binding to proteins, or translating into proteins, thus affecting GC growth, metastasis, and therapy resistance. For instance, circAKT3 is highly expressed in drug-resistant GC cells and sponges miR-198 to mediate the resistance to cisplatin [13]. The upregulation of circCUL3 regulates miR-515-5p/STAT3/HK2 signaling axis to accelerate the Warburg effect and promote GC progression [14]. Increased expression level of circOSBPL10 is suggested as a prognostic marker of the overall survival and disease-free survival of GC patients [15]. These findings suggest that circRNAs are important regulators of GC progression and further study of circRNAs would provide new understanding of GC pathogenesis and targets for GC diagnosis and therapy.

In this study, we identified a previously unknown circRNA transcribed from human *DIDO1* (death-inducer obliterator 1) gene (termed as circDIDO1) and explored the functional roles, underlying mechanism, and clinical implications of circDIDO1 in GC. We reported that circDIDO1 exerted a tumor suppressor role by encoding a novel 529 aa protein (DIDO1-529aa) to inhibit PARP1 activity and binding to PRDX2 protein to promote its degradation. Decreased expression of circDIDO1 in tumor tissues indicated adverse clinical outcome of GC patients and overexpression of circDIDO1 inhibited GC growth and metastasis in mouse models.

## Methods

### Bioinformatic analysis of circRNA expression profile in Gene Expression Omnibus datasets

Microarray data was downloaded from the Gene Expression Omnibus (GEO) datasets and DESeq2 package was used to analyze differentially expressed circRNAs. Fold change ≥ 2 and *p* value < 0.05 were set as the threshold for significantly differential expression.

### Patients and tissue samples

A total of 102 paired GC and adjacent non-cancerous tissues (5 cm away from the tumor edge) were obtained from Department of General Surgery, the Affiliated People’s Hospital of Jiangsu University. Written informed consent was obtained from all the patients and this study was approved by the Institutional Ethical Committee of Jiangsu University. All of the tissues were frozen in liquid nitrogen and then stored at -80 °C for further use. The patients included in this study had not received any preoperative therapies.

### RNA immunoprecipitation (RIP) assay

RIP assay was performed by using Magna RIP RNA-Binding Protein Immunoprecipitation Kit (Millipore, Bedford, MA). Cells were lysed in lysis buffer containing a protease inhibitor cocktail and RNase inhibitor. Magnetic beads were pre-incubated with the anti-Ago2 antibody or anti-mouse IgG for 1 h at room temperature, and lysates were immunoprecipitated with beads at 4 °C overnight. RNA was purified and subjected to qRT-PCR analysis.

### Tagged RNA affinity purification (TRAP) assay

Control and circDIDO1 overexpressing vectors that contain the stem-loop structure of MS2 (MS2 and circRNA-MS2) and GST-MS2 overexpressing vector were constructed by Biosense (Guangzhou, China). MS2 and circRNA-MS2 vectors were co-transfected with GST-MS2 into GC cells to obtain the GST-MS2-circRNA complex. Then, the complex was pulled down by glutathione magnetic beads. The circRNA-binding proteins were identified by mass spectrometry and validated by Western blot.

### RNA sequencing

Total RNA were extracted from control and circDIDO1 overexpressing GC cells and sent for sequencing by Illumina HiSeq sequencer (Cloundseq, Shanghai, China). Cutadapt, Hisat2, and Cuffdiff software were used to compare high-quality reads to the genome, obtain the FPKM value, and calculate the differentially expressed genes between control and circDIDO1 overexpression groups. The heatMap2 function in the R package was used for cluster analysis of differentially expressed mRNAs with FPKM values.

### LC–MS/MS

The proteases-digested protein samples were analyzed by liquid-mass spectrometry (LC–MS/MS) to obtain a raw file of the original mass spectrometry results. Byonic software was used to analyze the raw file and search the uniprot-Homo sapiens data to obtain the identified protein results.

### Co-immunoprecipitation (Co-IP) assay

Antibody and magnetic beads were cross-linked by disuccinimidyl suberate (DSS). Cells were lysed by Pierce immunoprecipitation lysis buffer supplemented with protease inhibitor cocktail (Thermo, Waltham, MA). After incubation with cell lysate at 4 °C overnight, the beads were washed with cell lysis buffer. The proteins were eluted from the magnetic beads for further analysis.

### Mouse tumor models

All the animal studies were approved by the Animal Care and Use Committee of Jiangsu University. BALB/c nude mice aged 4–6 weeks were purchased from the Model Animal Research Center of Nanjing University and maintained in accordance with the institutional policies. Vector or circDIDO1 transfected HGC-27 cells were collected in PBS, subcutaneously injected into the mice to construct subcutaneous xenograft tumor models (5 × 10^6^ cells per mouse; *n* = 5/group), and intraperitoneally injected into mice to construct peritoneal metastasis models (5 × 10^6^ cells per mouse; *n* = 5/group). The protocol was approved by the Animal Use and Care Committee of Jiangsu University.

### Statistical analysis

All statistical data was analyzed by using SPSS software 16.0 (SPSS, Chicago, IL) and GraphPad Prism 7.0 (GraphPad Software, La Jolla, CA). Student’s *t*-test and one way ANOVA test were used according to actual conditions. Survival analysis was performed using the Kaplan–Meier method and log-rank test for survival curves. The value of *P* < 0.05 was regarded as statistically significant.

## Results

### CircDIDO1 is downregulated in GC and its low level predicts poor prognosis

**Fig. 1:**
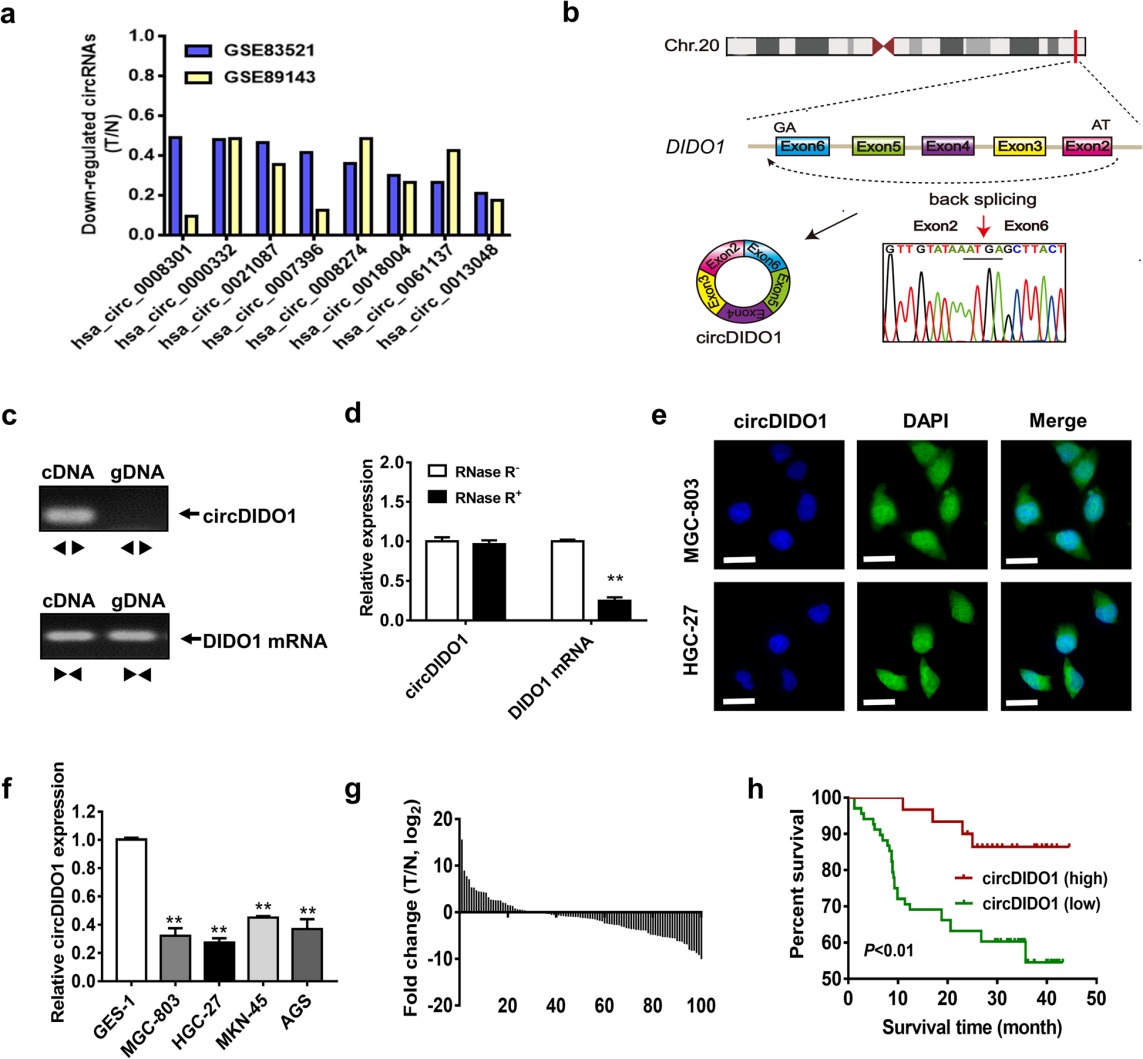
CircDIDO1 is identified as a circRNA downregulated in GC. **a**) GEO datasets (GSE83521 and GSE89143) were downloaded for integrated analyses of differentially expressed circRNAs. The common downregulated circRNAs were listed as indicated. **b**) The annotated region in *DIDO1* gene for the formation of circDIDO1 was shown. The exact sequence of back splicing site in circDIDO1 was confirmed by sequencing. **c**) PCR detection of circDIDO1 and linear transcript of DIDO1 by divergent and convergent primers in cDNA and gDNA of GC cells. **d**) The stability of circDIDO1 and DIDO1 mRNA was detected by RNase R degradation assay. Data are shown as means ± SD (*n* = 3, **P < 0.01). **e**) The distribution of circDIDO1 in GC cells was examined by RNA-FISH with a specific probe. Cell nuclei were counterstained with DAPI. Scale bars = 25 μm. **f**) The expression levels of circDIDO1 in GC cells (SGC-7901, HGC-27, MGC-803, AGS, and MKN-45) and normal gastric mucosa epithelial cells (GES-1). Data are shown as means ± SD (*n* = 3, **P < 0.01). **g**) The expression levels of circDIDO1 in tumor and matched non-tumor tissues from 102 GC patients were analyzed by qRT-PCR. **h**) The association between circDIDO1 expression level and overall survival time was analyze by Kaplan–Meier plot. Log-rank tests were used to determine statistical significance

To identify new circRNAs in GC, we extracted circRNA microarray data (GSE83521 and GSE89143) from Gene Expression Omnibus (GEO, https://www.ncbi.nlm.nih.gov/geo/) to analyze circRNA expression profiles [16, 17]. By intersecting differentially expressed circRNAs from these two datasets, we identified 8 circRNAs that were significantly downregulated in GC tissues compared to adjacent normal tissues (Fig. 1a). Considering the relative expression level and detection specificity, we chose hsa_circ_0061137 as the target for next study. Hsa_circ_0061137 is formed by back-splicing of exons 2–6 of the linear transcript of *DIDO1* (death-inducer obliterator 1) gene with a length of 1787 nucleotides (thus termed as circDIDO1) (Fig. 1b). PCR results showed that circDIDO1 could be amplified by divergent primers from cDNA but not gDNA of GC cells while the linear transcript could be amplified by convergent primers from both (Fig. 1c). In accordance, sequencing results confirmed the existence of back-splicing site in divergent primers-amplified PCR product (Fig. 1b). RNase R degradation assay results showed that the linear transcript of *DIDO1* was degraded by RNase R treatment while circDIDO1 was resistant to this treatment (Fig. 1d). RNA-FISH assay results revealed that circDIDO1 distributed in both the nucleus and the cytoplasm of GC cells (Fig. 1e). In summary, these results indicate that circDIDO1 is a newly discovered circular RNA in GC.

We then detected the expression of circDIDO1 in human GC cells and tissues. QRT-PCR results showed that circDIDO1 was significantly downregulated in GC cells compared to normal gastric mucosa epithelial cells (Fig. 1f). In addition, we examined the expression of circDIDO1 in 102 paired tumor tissues and normal tissues from GC patients and found that circDIDO1 expression was significantly downregulated in tumor tissues of GC patients compared to paired normal tissues (Fig. 1g). CircDIDO1 expression levels were inversely correlated with tumor size and distant metastasis (Table S1). Moreover, the GC patients who had low levels of circDIDO1 came out with a notably shorter survival time than those who had high ones (Fig. 1h). Collectively, these results indicate that circDIDO1 is lowly expressed in GC and its downregulation predicts poor prognosis.

### CircRNA exerts tumor suppressive roles in GC

**Fig. 2:**
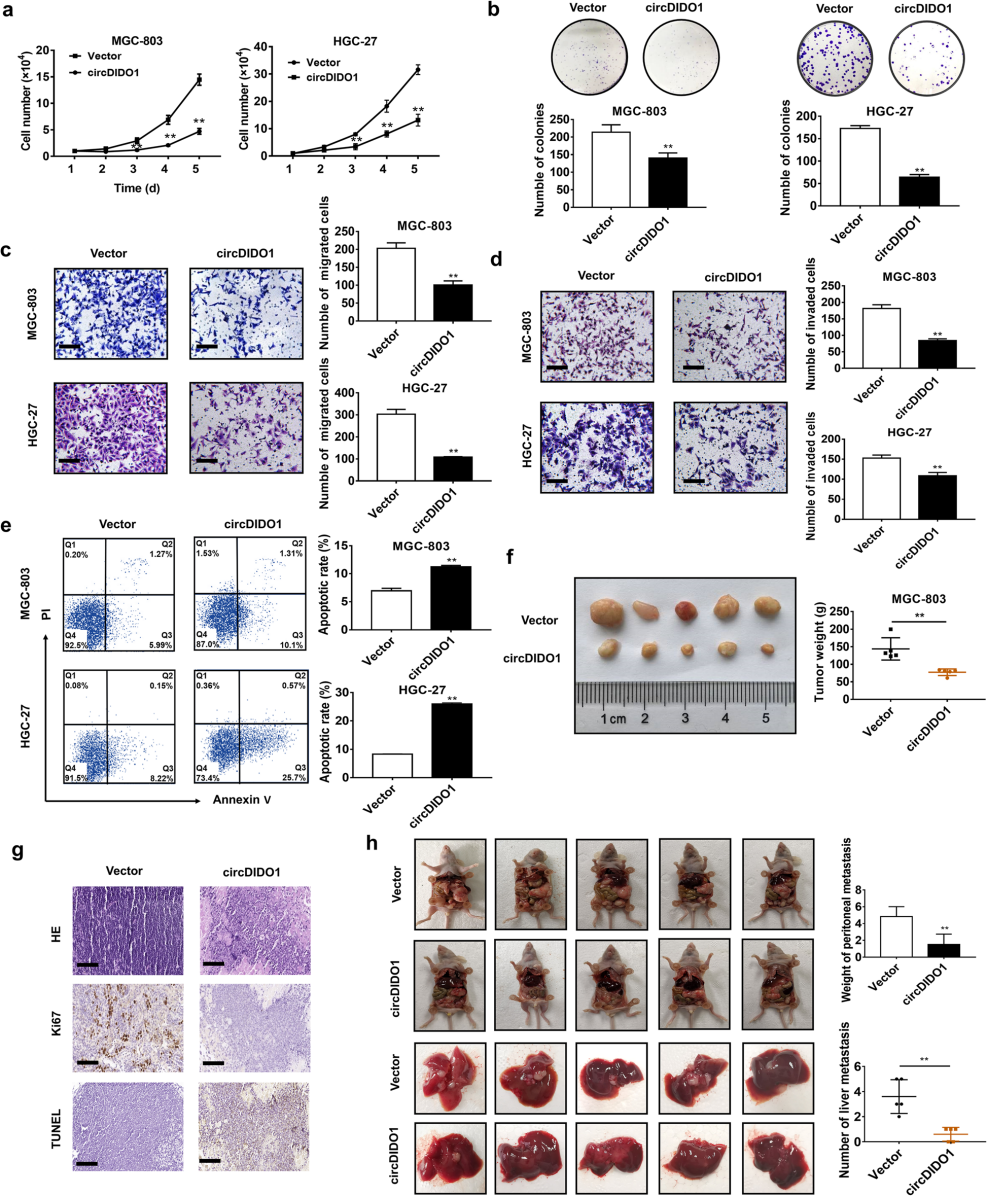
CircDIDO1 overexpression inhibits GC growth and metastasis in vitro and in vivo. **a**–**e**) Cell growth curve (**a**), colony formation (**b**), transwell migration (**c**), matrigel invasion assays (**d**) and flow cytometry analyses (**e**) for control and circDIDO1 overexpressing GC cells. Data are shown as means ± SD (*n* = 3, **P < 0.01). Scale bars = 100 μm. **f**) Balb/c nude mice were subcutaneously injected with control or circDIDO1 overexpressing HGC-27 cells. The size and weight of xenograft tumors were shown. Data are shown as means ± SD (*n* = 5 for each mice group, **P < 0.01). **g**) HE, Ki-67 and TUNEL staining of xenograft tumor tissues. Scale bars = 100 μm. **h**–**i**) Balb/c nude mice were intraperitoneally injected with control or circDIDO1 overexpressing HGC-27 cells. The number of peritoneal metastatic tumors and liver metastatic tumors was shown. Data are shown as means ± SD (*n* = 5 for each group, **P < 0.01)

We next explored the biological roles of circDIDO1 in GC by gain- and loss-of-function studies. The results of cell growth, colony formation, transwell migration, matrigel invasion assays and flow cytometry analyses showed that circDIDO1 overexpression significantly inhibited the proliferation (Fig. 2a and 2b), migration (Fig. 2c), and invasion (Fig. 2d) abilities of GC cells while promoted their apoptosis (Fig. 2e). In contrast, circDIDO1 knockdown had the opposite effects (Figure S1).

We then evaluated the effect of circDIDO1 on GC growth in mouse models. As shown in Fig. 2f, the size and weight of tumors in circDIDO1 overexpressing group were significantly lower than those in control group at 30 days after inoculation. Immunohistochemical staining results revealed that the percentage of Ki-67-positive proliferating cells decreased while that of TUNEL-positive apoptotic cells increased in circDIDO1 overexpressing group compared to control group (Fig. 2g).

To analyze the effect of circDIDO1 on GC metastasis, we established a peritoneal metastasis model by injecting control and circDIDO1 overexpressing cells into the abdominal cavity of nude mice. Four weeks later, the nude mice were sacrificed and the abdominal metastases and liver metastases were observed. Compared with control group, circDIDO1 overexpressing group had significantly smaller volume and weight of mesenteric tumors (Fig. 2h). In accordance, the number of liver metastases in circDIDO1 overexpressing group was significantly lower than that in control group (Fig. 2h). Immunohistochemical staining results showed that the expression of EMT markers E-cadherin was significantly up-regulated while that of N-cadherin was down-regulated in liver metastasis tissues in circDIDO1 overexpressing group compared to control group (Figure S2a).

### CircDIDO1 encodes a 529aa tumor suppressor protein

**Fig. 3:**
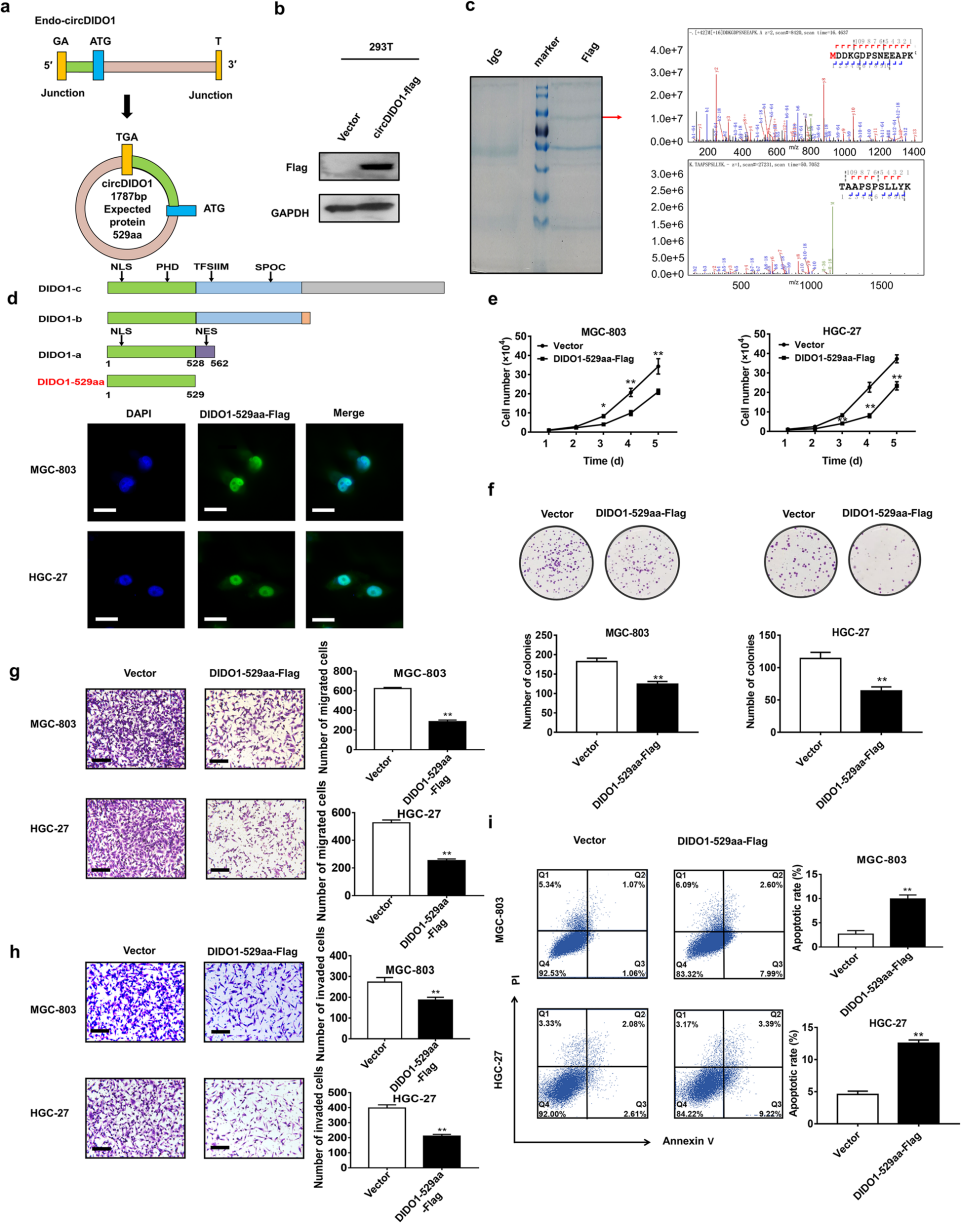
CircDIDO1 encodes a novel 529 aa protein. **a**) The predicted putative open reading frame (ORF) in circDIDO1. **b**) Detection of the expression of nascent protein encoded by circDIDO1 overexpressing vector with Flag tag (circDIDO1-Flag) by Western blot with Flag antibody. **c**) The nascent protein was purified from circDIDO1-Flag transfected 293 T cells by Co-IP with Flag antibody and separated by SDS-PAGE. The image of Coomassie brilliant blue staining for the gel was shown. The nascent protein was harvested for LC–MS/MS analysis to identify its peptide sequence. The front amino acid sequence (upper right) and the end amino acid sequence (lower right) were shown. **d**) Schematic diagram of domains of DIDO1 protein isoforms and DIDO1-529aa. The subcellular distribution of DIDO1-529aa in GC cells was examined by immunofluorescence. Scale bars = 25 μm. **e**–**i**) Cell growth curve (**e**), colony formation (**f**), transwell migration (**g**), matrigel invasion assays (**h**) and flow cytometry analyses of apoptosis (**i**) in control or linear form vector of DIDO1-529aa (DIDO1-529aa-Flag) overexpression GC cells. Data are shown as means ± SD (*n* = 3, **P < 0.01, *P < 0.05)

Our bioinformatic analysis by circRNADb software showed that circDIDO1 has ribosome entry site (IRES), open reading frame (ORF), and m6A modification (Figure S3a), suggesting that it may have a potential to encode protein. The reverse splicing site of circDIDO1 forms a stop codon and the predicted ORF of circDIDO1 is supposed to encode a putative 529 aa protein (Fig. 3a), which was termed as DIDO1-529aa. When detected by an antibody against DIDO1 protein, we found that circDIDO1 overexpression generated a novel protein with a molecular weight close to that of DIDO1 (Figure S3b). CircDIDO1 overexpression had no effect on DIDO1 gene expression (Figure S3c). To confirm that circDIDO1 has protein-encoding ability, we constructed a circDIDO1 overexpressing vector with FLAG tag (circDIDO1-Flag) before the last nucleotide of the entire sequence (i.e., the predicted stop codon of circDIDO1) and transfected it into 293 T cells. Western blot results showed that FLAG antibody could detect the expression of nascent protein, which indicates that circDIDO1 has protein-encoding ability (Fig. 3b). Subsequently, we purified the nascent protein by IP with anti-FLAG antibody for further validation. The results of LC–MS/MS showed that the amino acid sequence of nascent protein completely matched with that of the predicted one, which confirmed that DIDO1-529aa was translated from circDIDO1 (Fig. 3c and Figure S3d).

We then compared the amino acid sequence of DIDO1-529aa with that of DIDO1 protein. We found that DIDO1-529aa shared similar amino acid sequence with DIDO1 protein isoform a (DIDO1-a). Compared with DIDO1-a, DIDO1-529aa lost the last 33 amino acids of C-terminus, which is the nuclear export sequence (NES) of DIDO1-a (Fig. 3d). We further determined the distribution of DIDO1-529aa in GC cells by immunofluorescence and found that DIDO1-529aa mainly localized in the nucleus (Fig. 3d). To further explore the function of DIDO1-529aa, we constructed the linear form of vector for DIDO1-529aa with Flag tag (DIDO1-529aa-Flag) and transfected it into GC cells. The results of functional assays showed that DIDO1-529aa overexpression inhibited GC cell proliferation, migration and invasion, but promoted GC cell apoptosis (Fig. 3e–i). In summary, these findings indicate that circDIDO1 encodes a 529aa protein with tumor suppressor activity.

### DIDO1-529aa interacts with PARP1 to inhibit its activity

**Fig. 4:**
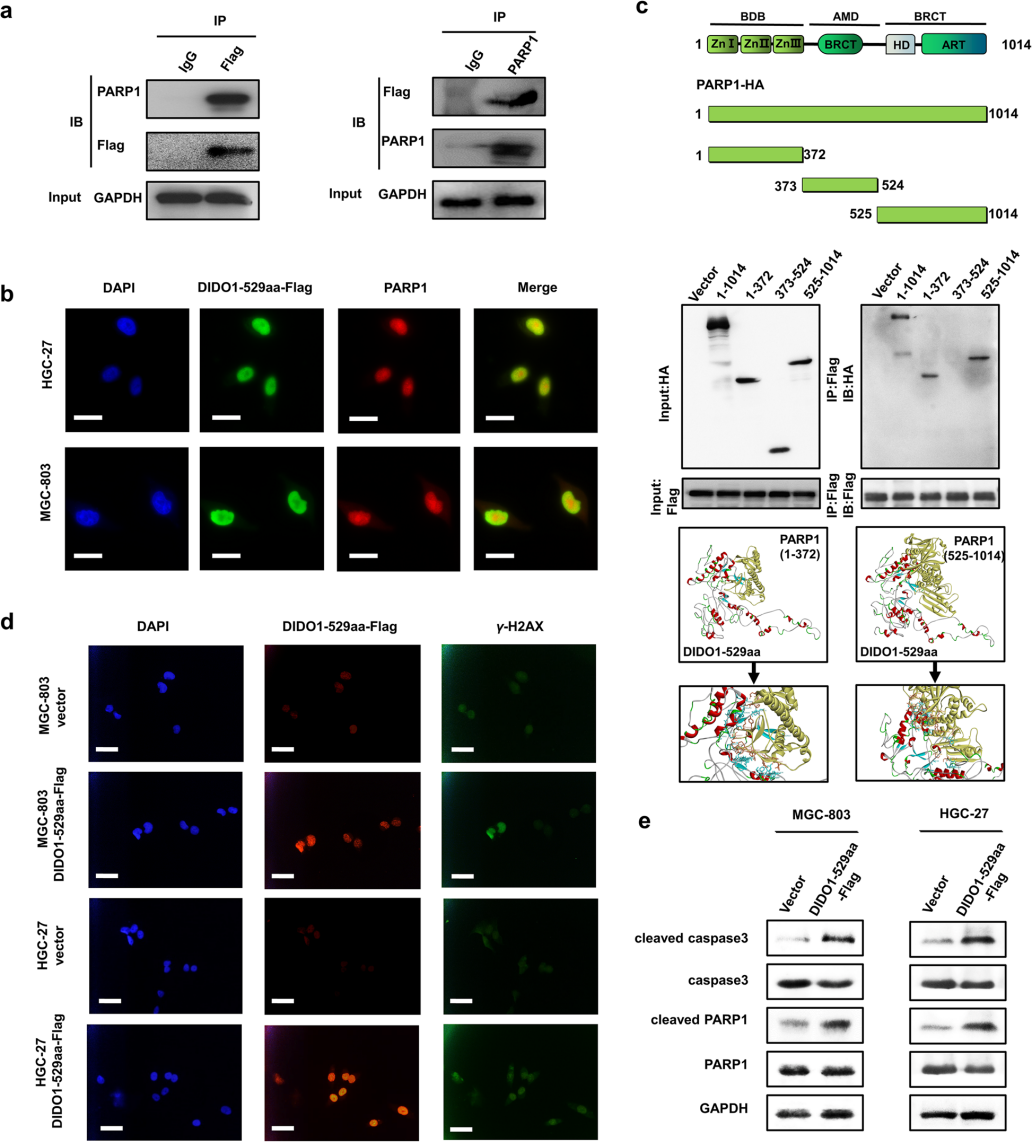
DIDO1-529aa interacts with PARP1. **a**) Co-IP analyses of the interaction between DIDO1-529aa and PARP1. **b**) Co-localization of DIDO1-529aa and PARP1 in the nucleus was examined by immunofluorescence. Scale bars = 25 μm. **c**) DIDO1-529aa-Flag vector was co-transfected with distinct HA-tagged PARP1 deletions (1–372, 373–524, and 525–1014 aa) into 293 T cells. The interaction of DIDO1-529aa with different PARP1 mutants was tested by Co-IP. DIDO1-529aa was marked in multiple colors and PRPA1 was marked in yellow. The bottom figure showed the structure of DIDO1-529aa-PARP1 interaction. The possible interacting amino acids in DIDO1-529aa were marked in cyan and those in PRPA1 marked in brown. **d**) Immunofluorescent staining of γ-H2AX in control or DIDO1-529aa overexpressing GC cells. Scale bars = 50 μm. **e**) Western blot analyses for the expression of cleaved PARP1 and caspase-3 in control and DIDO1-529aa overexpression GC cells

To understand how DIDO1-529aa exerts its function in GC cells, we carried out Co-IP assay with anti-Flag antibody and performed LC–MS/MS analysis. The results showed that poly (ADP-ribose) polymerases-1 (PARP1) was among the proteins that were co-immunoprecipitated by DIDO1-529aa. The binding of DIDO1-529 and PARP1 was further validated by Co-IP and Western blot (Fig. 4a). In addition, immunofluorescence results showed that DIDO1-529aa co-localized with PARP1 in the nucleus of GC cells (Fig. 4b). PARP1 protein has three domains, namely DNA binding domain (DBD), automodification domain (AMD) and catalytic domain (CAT). We then constructed three truncated PARP1 overexpression vectors with HA tag according to the structure of PARP1 protein (Fig. 4c) and co-transfected them with DIDO1-529aa-Flag vector into 293 T cells. Co-IP results showed that DIDO1-529aa interacted with both 1–372 aa and 525–1014 aa domains of PARP1 protein (Fig. 4c). Furthermore, we used I-TASSER software to predict the conformation of DIDO1-529aa and docked it with PRAP1 protein by ZDOCK software. The most probable docking mode and the main interface residues for protein–protein interaction were shown in Fig. 4c. We further determined whether binding to DIDO1-529aa affects PARP1 activity in GC cells. Immunofluorescent staining results showed that the levels of DNA damage marker γ-H2AX were significantly higher in DIDO1-529aa overexpressing group than control group (Fig. 4d). In accordance, Western blot results revealed that DIDO1-529aa overexpression resulted in increased levels of cleaved caspase 3 and cleaved PARP1 in GC cells (Fig. 4e), both of which are markers for cells undergoing apoptosis.

**Fig. 5:**
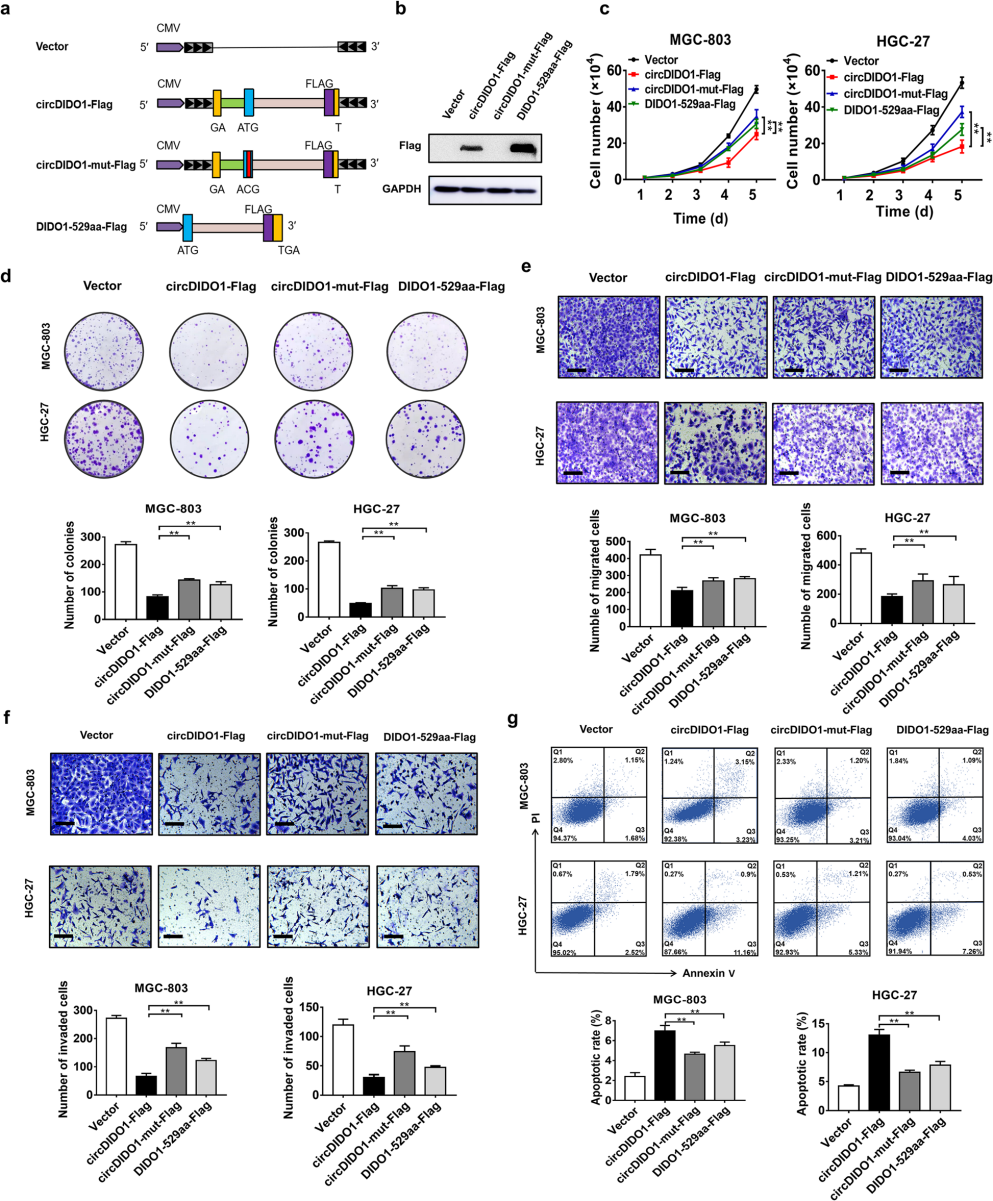
CircDIDO1 exerts tumor suppressor function in GC through dual mechanisms. **a**) Schematic diagram of mutant circDIDO1-Flag vector (circDIDO1-mut-Flag) that lacks protein-coding ability. **b**) Western blot assays for the expression of DIDO1-529aa in 293 T cells transfected with control, circDIDO1-Flag, circDIDO1-mut-Flag, and DIDO1-529aa-Flag vectors (detected by Flag antibody). Cell growth curve (**c**), colony formation (**d**), transwell migration (**e**), matrigel invasion (**f**) assays and flow cytometry analysis of apoptosis (**g**) in control, wild-type or mutant circDIDO1-Flag vector, and DIDO1-529aa-Flag vectors transfected GC cells. Data are shown as means ± SD (*n* = 3, **P < 0.01)

We then constructed a mutant circDIDO1 overexpressing vector (circDIDO1-mut-Flag) that lacks the ability to encode DIDO1-529aa protein and compared its function with that of wild-type circDIDO1 (circDIDO1-Flag) and linear form of DIDO1-529aa (DIDO1-529aa-Flag) vectors (Fig. 5a). Western blot results showed that both circDIDO1-Flag and DIDO1-529aa-Flag could generate DIDO1-529aa protein as detected by Flag antibody; however, this role was lost in circDIDO1-mut-Flag group (Fig. 5b). The results of functional assays showed that circDIDO1-Flag vector displayed the most significant role in inhibiting the proliferation (Fig. 7c,d), migration (Fig. 5e), and invasion (Fig. 5f) of GC cells and promoting apoptosis in GC cells (Fig. 5g). In contrast, these effects were partially weakened in circDIDO1-mut-Flag and DIDO1-529aa-Flag groups, suggesting that circDIDO1 may inhibit GC progression through multiple mechanisms.

### CircDIDO1 interacts with PRDX2 protein

**Fig. 6:**
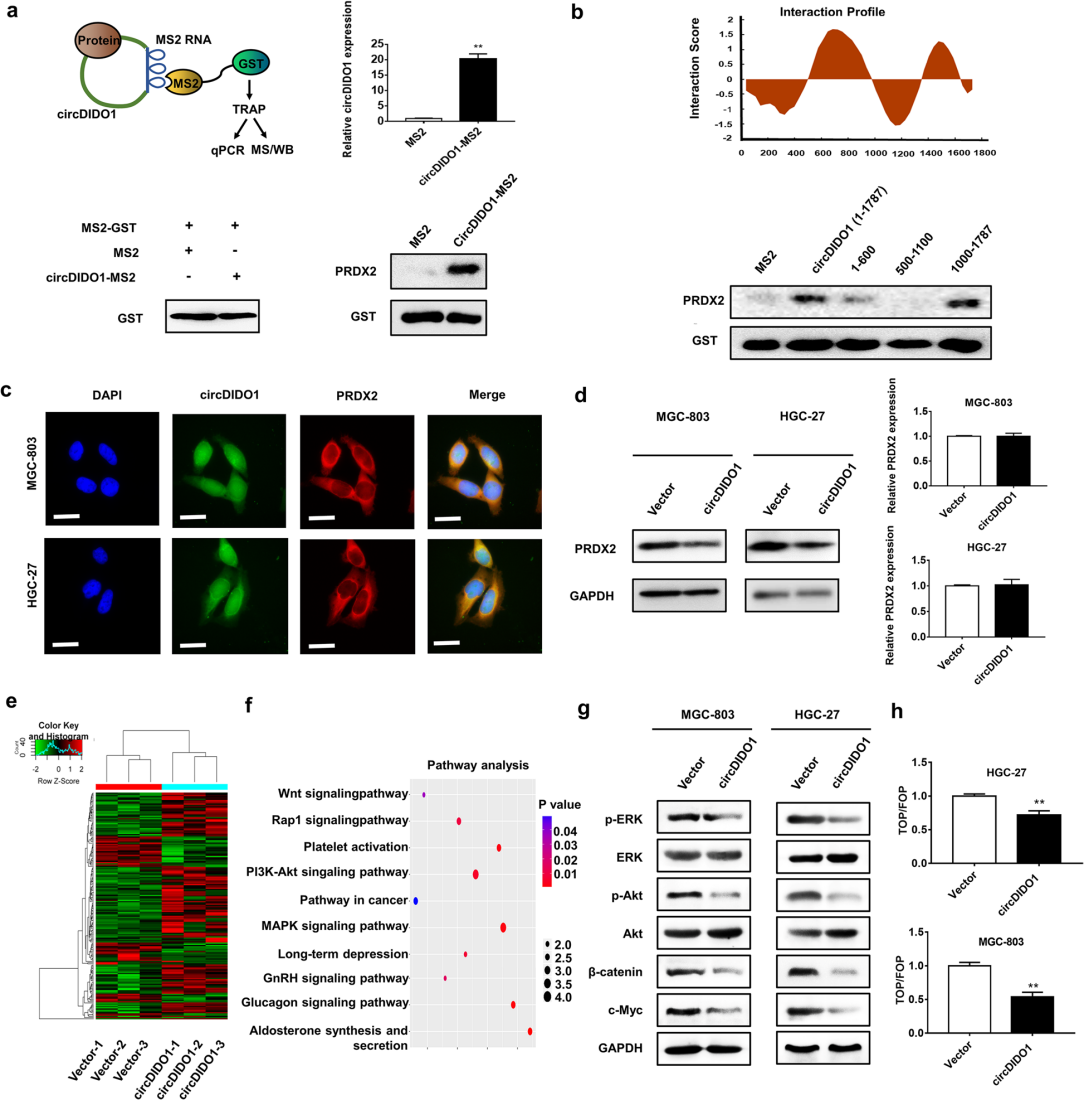
CircDIDO1 interacts with PRDX2 protein in GC cells. **a**) circDIDO1-binding proteins were identified by TRAP experiment and LC–MS/MS analysis. GST protein expression was detected by Western blot and circDIDO1 expression was detected by qRT-PCR. The binding of circDIDO1 to PRDX2 protein was validated by TRAP experiment followed by Western blot. **b**) The potential binding site of PRDX2 in circDIDO1 was predicted by catRAPID and verified by Western blot. **c**) Co-localization of circDIDO1 and PRDX2 protein in GC cells was determined by RNA-FISH and immunofluorescence. Scale bars = 25 μm. **d**) Western blot and PCR analyses of PRDX2 expression in control or circDIDO1 overexpressing GC cells. **e**) Heatmap of differentially expressed genes in control and circDIDO1 overexpressing HGC-27 cells. **f**) Enrichment of differentially expressed genes in different pathways by Pathway analysis. **g**) The expression of proteins associated with PRDX2 downstream signaling pathways in control and circDIDO1 overexpressing GC cells were detected by Western blot. **h**) TOP/FOP flash luciferase assay for control and circDIDO1 overexpressing GC cells. Data are shown as means ± SD (*n* = 3, **P < 0.01)

To depict other mechanisms that are responsible for the roles of circDIDO1 in GC, we carried out a tagged RNA affinity purification (TRAP) assay to identify its interacting proteins (Fig. 6a). CircDIDO1-MS2 vector was co-transfected with GST-MS2 vector into HGC-27 cells. The potential binding proteins of circDIDO1 were obtained by GST pull-down and identified by LC–MS/MS analysis. We found that 27 proteins were significantly enriched in circDIDO1 group and seven of them were only detectable in circDIDO1 group but not in control group, including PRDX2, S100A9, and DDX42, among others (Table S2). Validation experiments by TRAP and Western blot showed that circDIDO1 had a strong binding ability to PRDX2 protein (Fig. 6a). We predicted the binding sequence for PRDX2 in circDIDO1 by catRAPID software (Fig. 6b, upper panel) and constructed three truncated circDIDO1 overexpressing vectors (1–600, 500–1100, and 1000–1787 nt). TRAP assay results showed that PRDX2 mainly bound to the 1000–1787 nt region of circDIDO1 (Fig. 6b, lower panel). In addition, cell distribution studies showed that circDIDO1 and PRDX2 had co-localization in the cytoplasm of GC cells (Fig. 6c). Moreover, we found that circDIDO1 overexpression decreased the protein level of PRDX2 while had no significant effect on its mRNA level (Fig. 6d). Gain- and loss-of-function studies revealed that PRDX2 knockdown inhibited while its overexpression promoted GC cell proliferation, migration, and invasion (Figure S4). Moreover, the results of rescue experiments showed that PRDX2 overexpression partially reversed the suppressive roles of circDIDO1 in GC cell proliferation, migration, and invasion (Figure S5), suggesting that PRDX2 is an important target of circDIDO1.

### CircDIDO1 disrupts PRDX2 downstream pathways in GC cells

To analyze the downstream signaling pathways that are regulated by circDIDO1, we carried out RNA sequencing for control and circDIDO1 overexpressing GC cells and performed cluster and pathway analyses for gene expression profiles (Fig. 6e,f). We found that 134 genes were up-regulated and 52 genes were down-regulated in circDIDO1 overexpression group compared to control group (Table S4). The altered expression of several identified genes in circDIDO1 overexpressing group was verified by qRT-PCR (Figure S6). Pathway analysis suggested that the differentially expressed genes were enriched in signaling pathways that are closely associated with cancer progression, such as Wnt/β-catenin, MAPK, and PI3k/Akt pathways (Fig. 6f). Previous studies demonstrate that PRDX2 protein is a critical regulator of these pathways. To this end, we examined whether circDIDO1 regulates these pathways through down-regulation of PRDX2 in GC cells. Western blot results confirmed that circDIDO1 overexpression inactivated these pathways in GC cells and tumor tissues (Fig. 6g and Figure S2b), which shares a similar change pattern to those observed in GC cells with PRDX2 knockdown. In particular, circDIDO1 overexpression inhibited the expression and activity of β-catenin in GC cells (Fig. 6h and Figure S7), which is in accordance with previous studies showing that PRDX2 is an important regulator of β-catenin signaling pathway in other cancer cells.

### CircDIDO1 promotes RBX1-mediated ubiquitination and degradation of PRDX2

**Fig. 7:**
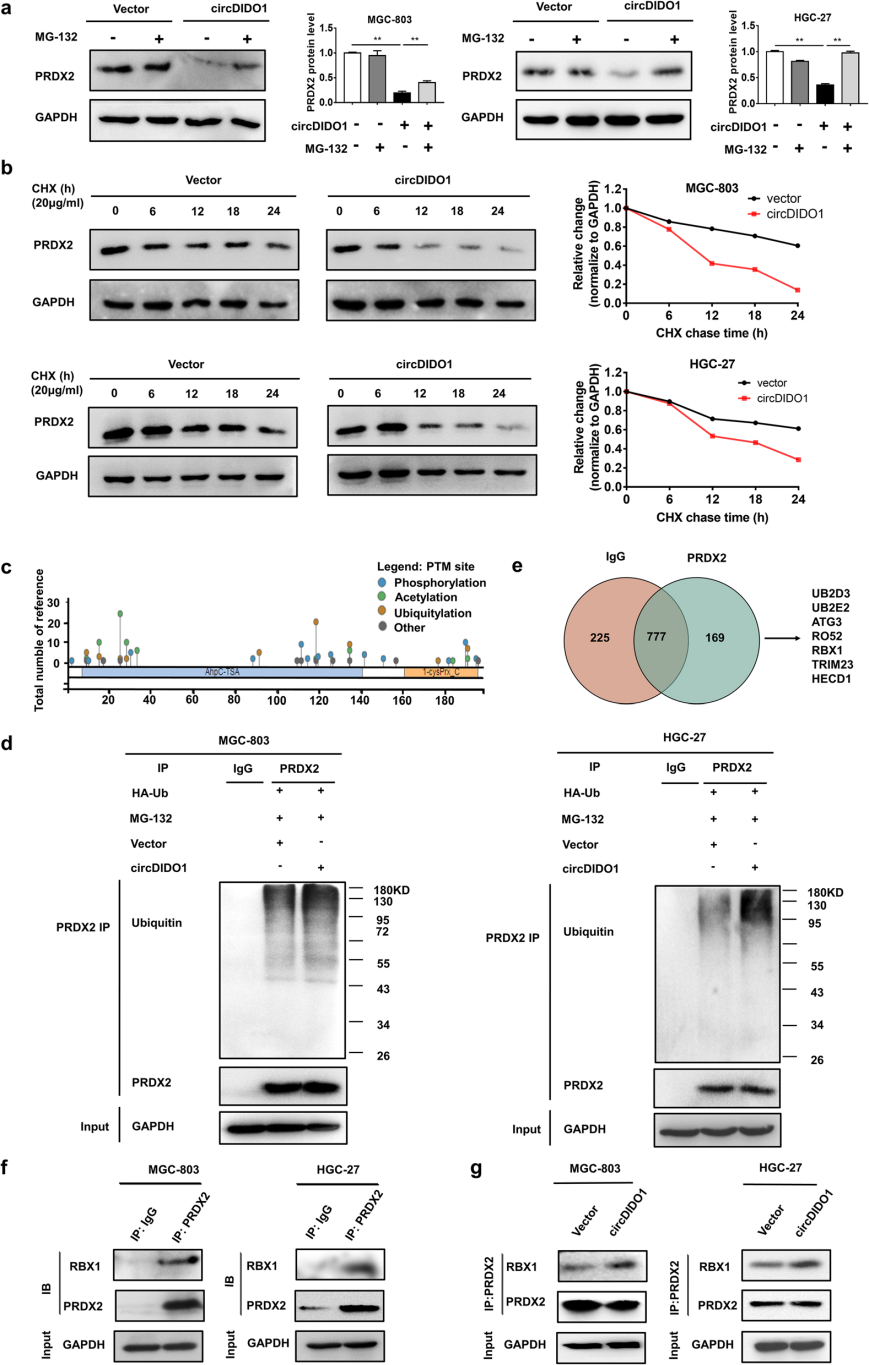
CircDIDO1 promotes PRDX2 ubiquitination and degradation by RBX1. **a**) Protein degradation was blocked with MG-132 (40 μM) for 8 h. PRDX2 protein levels in GC cells with or without circDIDO1 overexpression were detected by Western blot. Data are shown as means ± SD (*n* = 3, **P < 0.01). **b**) Protein biosynthesis in GC cells was blocked with 20 μg/mL of cycloheximide (CHX). PRDX2 protein levels in GC cells with or without circDIDO1 overexpression at different time points were examined by Western blot. **c**) Prediction of ubiquitination sites in PRDX2 protein by PhosphoSitePlus software. **d**) Venn plot for the potential interacting proteins of PRDX2 as identified by Co-IP and LC–MS/MS. **e**) GC cells were transfected with ubiquitin (Ub) and circDIDO1 and treated with MG-132. Whole cell lysates were immunoprecipitated with PRDX2 antibody followed by detection with ubiquitin antibody. **f**) Validation of the interaction between PRDX2 and RBX1 in GC cells by Co-IP. **g**) The interaction between PRDX2 and RBX1 in control and circDIDO1 overexpressing GC cells was examined by Co-IP and Western blot

We further performed MG-132 assay to determine whether circDIDO1 regulates PRDX2 protein stability. We found that pre-treatment with MG-132 prevented circDIDO1 overexpression-induced degradation of PRDX2 protein in GC cells (Fig. 7a). In consistent with this, cycloheximide (CHX) assay results showed that the half-life of PRDX2 protein in circDIDO1 overexpressing group was shorter than that in control group (Fig. 7b), suggesting that circDIDO1 accelerates PRDX2 protein degradation in GC cells.

To analyze whether circDIDO1 promotes PRDX2 protein degradation through ubiquitination-dependent mechanism, we predicted the potential ubiquitin modification sites in PRDX2 protein by PhosphoSitePlus software. We found that PRDX2 protein has multiple ubiquitin modification sites (Fig. 7c). We then co-transfected circDIDO1 with ubiquitin into GC cells and treated the transfected cells with MG-132. The results of Co-IP assay showed that circDIDO1 overexpression remarkably increased the levels of ubiquitinated PRDX2 protein (Fig. 7d). We then conducted Co-IP and mass spectrometry analyses to detect PRDX2-binding proteins and found that PRDX2 bound to a series of ubiquitin ligases (Fig. 7e and Table S4). We confirmed that PRDX2 bound to RBX1, an E3 ligase of SCF ubiquitination complex (Fig. 7f) and circDIDO1 overexpression significantly promoted the interaction between PRDX2 and RBX1 proteins (Fig. 7g). Collectively, these data suggest that circDIDO1 promotes RBX1-meidated PRDX2 ubiquitination and degradation in GC cells.

## Discussion

CircRNAs are key players in the pathogenesis of various diseases including cancer [18–20]. In this study, we identified a novel circRNA, circDIDO1, that has potent tumor suppressor activities in GC. We found that circDIDO1 expression was downregulated in the tumor tissues of patients with GC. Low levels of circDIDO1 were associated with larger tumor size and distal metastasis and indicated poor prognosis, suggesting that circDIDO1 may be utilized as a potential prognostic marker for GC. We revealed that circDIDO1 performed its tumor suppressor activity through dual mechanisms. First, circDIDO1 encoded a 529aa protein that interacted with PARP1 to inhibit its activity. Second, circDIDO1 bound to PRDX2 protein and promoted its degradation by RBX1-mediated ubiquitination. In addition to serve as miRNA sponge [21], recent studies have shown that circRNAs can also bind to proteins and even translate into proteins [22, 23]. Our findings provide new evidence for the potent roles of circRNAs in cancer. Moreover, circDIDO1 overexpression efficiently inhibited GC growth and metastasis in mouse models, indicating that circDIDO1 may also be a potential target for GC therapy.

The *DIDO* gene encodes three splicing variants (Dido1, Dido2, and Dido3 in mice; DIDO1-a, DIDO1-b, and DIDO1-c in human) and has been implicated in apoptosis and embryonic stem cell self-renewal [24, 25]. Previous studies suggest a tumor suppressor role of *DIDO* gene in mice and demonstarte that targeted loss of *DIDO* gene causes myeloid neoplasms [26]. *DIDO* expression is reduced in some patients with myeloid neoplasms compared to healthy controls [26]. DIDO1-a, the shortest splicing variant encoded by the *DIDO* gene, has been reported to regulate programmed cell death [27]. Under physiological conditions, DIDO1-a mainly locates in the cytoplasm. After starvation or oncogene-induced apoptosis, it translocates to the nucleus, where it upregulates caspase levels and activities [28]. The molecular mechanisms by which DIDO1 induces apoptosis are not fully understood. We found that a circular form of *DIDO1*, circDIDO1, encoded a new DIDO1 protein isoform that lacked the nuclear export sequence of DIDO1-a protein and persistently located in the nucleus, where it interacted with PARP1 protein to inhibit its DNA repair ability. In addition, we also found that circDIDO1 bound to PRDX2 protein and induce its degradation through ubiquitination-proteasome system. Our results provide new insights into the mechanism for the function of *DIDO1* gene, since PARP1 and PRDX2 have been known to be closely associated with cell apoptosis. Further studies are needed to clarify the fine-tuned regulation of *DIDO1* gene expression in normal cells and its role in the development and progression of human cancers.

PARP is a family of multifunctional nuclear proteins that participate in the synthesis of poly ADP-ribose and are involved in genomic stability, DNA damage response, apoptosis, transcriptional regulation, and chromatin remodeling [29]. PARP-1 is the first discovered and most studied PARP family member with core DNA repair function. When DNA single strand break occurs in the cell, PARP-1 actively participates in the repair process through multiple mechanisms. PARP-1 recognizes single strand break in DNA by DBD domain and modify the receptor protein to adjust its conformation, stability, and activity by CAT domain, which are both important for the repairing process. Inhibition of PARP-1 impairs DNA damage repair and causes apoptosis of cancer cells. Therefore, PARP1 has become a popular target in the field of cancer therapy and PARP-1 inhibitors have been suggested to be effective to potentiate both chemotherapy and radiotherapy. Although several PARP1 inhibitors have been approved, they encounter problems such as low selectivity and therapy resistance when used in the clinic [30]. PARP-1 inhibitors mainly function by inhibiting the catalytic activity of PARP-1 [31]. Herein, we found that DIDO1-529aa simultaneously bound to the DBD and CAT domains of PARP1. Moreover, we observed that DIDO1-529aa overexpression increased DNA damage and induced apoptosis in GC cells. The interaction of DIDO1-529aa with PARP1 may not only inhibit the binding of PARP1 to damaged DNA, but also inhibit the enzymatic activity of PARP1, which provides a new direction for the design of PARP1 inhibitors with improved therapeutic efficacy.

PRDX2 is an antioxidant enzyme of the peroxiredoxin family and plays an important role in scavenging H_2_O_2_ and ROS levels, therefore protecting cells from oxidative stress. Previous studies demonstrate that PRDX2 is overexpressed in a variety of cancers and participates in multiple processes of cancer progression [32]. In addition to its antioxidant capacity, PRDX2 have functions in cell cycle regulation, apoptosis, gene expression and signal transduction [33, 34]. For instance, PRDX2 enhances the proliferation and tumorigenicity of hepatoma cells by regulating the ERK/FoxM1/cyclin D1 signal axis [35]. PRDX2 knockdown leads to the degradation of β-catenin and ultimately the inhibition of colon cancer cell growth [36]. A high level of PRDX2 has been demonstrated as a poor prognostic marker in cisplatin-resistant gastric cancer [37]. In this study, we found that circDIDO1 specifically bound to PRDX2 protein to inhibit its expression and thus inactivate its downstream signaling pathways, leading to the suppression of GC cell growth and aggressiveness. We reported that PRDX2 protein was degraded by a ubiquitination-dependent mechanism. Our results unraveled that circDIDO1 promoted the interaction between PRDX2 and RBX1 proteins, leading to the degradation of PRDX2 by proteasome. However, the detailed mechanism by which circDIDO1 promoted the binding between PRDX2 and RBX1 is currently unknown and deserves further investigation.

**Fig. 8:**
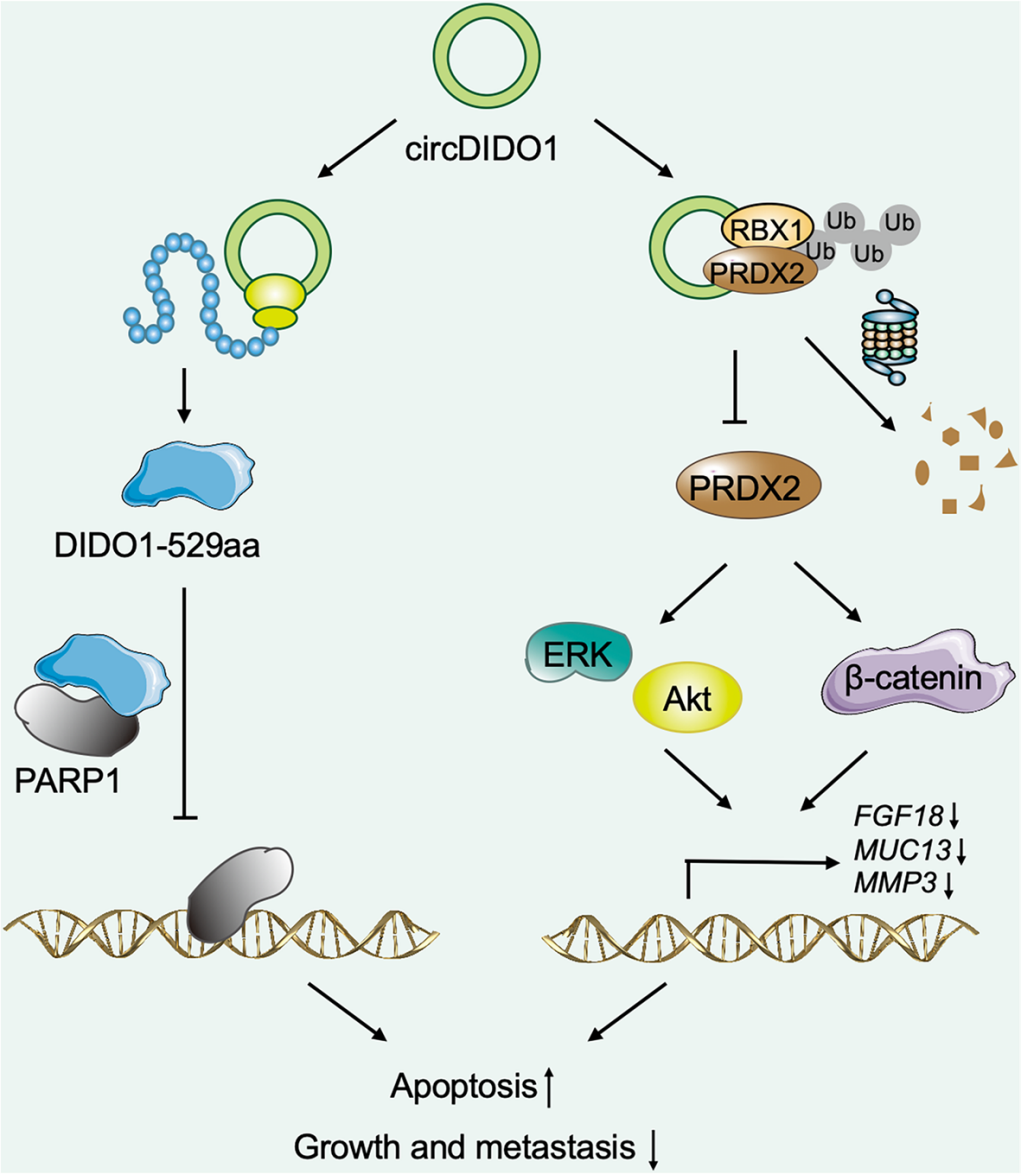
Proposed model for the mechanism of action of circDIDO1 in GC. CircDIDO1 is a new tumor suppressive circRNA in GC. circDIDO1 inhibits GC growth and aggressiveness by encoding a DIDO1-529aa protein to inhibit PARP1 activity and promoting RBX1-meidated ubiquitination and degradation of PRDX2

Conclusively, we reported the identification of a new circRNA that has potent tumor suppressor activities in GC (Fig. 8). We demonstrated that circDIDO1 inhibited the growth and aggressiveness of GC cells by encoding a DIDO1-529aa protein as PARP1 inhibitor and promoting the ubiquitination and degradation of PRDX2. Our study expands the understanding of circRNA function in GC pathogenesis and suggests a novel circRNA as potential biomarker and therapeutic target for CC.

## Supplementary Information

**Additional file 1: Figure S1**. CircDIDO1 knockdown promotes GC cell proliferation, migration, and invasion in vitro. a) Efficiency of gene knockdown in GC cells by siRNAs was confirmed by qRT-PCR. b) Cell growth curve, c) colony formation, d) transwell migration, and e) matrigel invasion assays for control and circDIDO1 knockdown GC cells.

**Additional file 2: Figure S2**. a) HE staining and immunohistochemical staining of EMT markers in liver metastasis tumors. Scale bars = 100 μm. b) The expression of proteins associated with PRDX2 downstream signaling pathways in the tumor tissues of control and circDIDO1 groups was detected by Western blot.

**Additional file 3: Figure S3**. Identification of circDIDO1-encoded 529aa protein. a) Prediction of the protein-encoding ability of circDIDO1 by circRNADb software. b) Western blot assays for predicted DIDO1-509aa protein expression in GC cells by DIDO1 antibody. c)qRT-PCR analysis of circDIDO1 and DIDO1 expression in control and circDIDO1 overexpressing GC cells. d) Protein sequencing result of DIDO1-529 aa.

**Additional file 4: Figure S4**. PRDX2 overexpression promotes while knockdown inhibits GC cell proliferation, migration, and invasion in vitro. a) Cell growth curve, b) colonyformation, c) transwell migration, and d) matrigel invasion assays for control and PRDX2 overexpressing GC cells. e) Cell growth curve, f) colony formation, g) transwell migration, and h) matrigel invasion assays for control and PRDX2 knockdown GC cells.

**Additional file 5: Figure S5**. PRDX2 partially rescues the inhibition of cell proliferation, migration, and invasion in circDIDO1 overexpressing GC cells. a) Cell growth curve, b)colony formation, c) transwell migration, and d) matrigel invasion assays for circDIDO1 overexpressing GC cells co-transfected with or without PRDX2.

**Additional file 6: Figure S6**. QRT-PCR analyses of differentially expressed genes in control and circDIDO1 overexpressing GC cells.

**Additional file 7: Figure S7**. PRDX2 knockdown inhibits β-catenin expression and activity in GC cells. a) Western blot assays for protein levels of β-catenin and its downstream targets in control and PRDX2 knockdown GC cells. b) TOP/FOP flash luciferase reporter assays for β-catenin activity in control and PRDX2 knockdown GC cells.

**Additional file 8: Table S1.** The relationship of circDIDO1 expression levels (ΔCt) in GC tissues with clinicopathological parameters.

**Additional file 9: Table S2.** The proteins enriched in circDIDO1-MS2 group.

**Additional file 10: Table S3.** The potential interacting proteins enriched in PRDX2 group.

**Additional file 11: Table S4.** The genes dysregulated in circDIDO1 overexpression cells (mRNA-seq).

**Additional file 12: Table S5.** Primer sequences for qRT-PCR.

**Additional file 13: Table S6.** The sequences of oligonucleotides.

**Additional file 14: Table S7.** The information of antibodies used in this study.

**Additional file 15: Supplementary Methods.**

## Data Availability

The datasets supporting the conclusions of this article are included within the article and its supplementary files.

## Abbreviations

AMD: Automodification domain
CAT: Catalytic domain
CHX: Cycloheximide
circRNAs: Circular RNAs
DAPI: 4’,6-Diamidino-2-phenylindole
DBD: DNA binding domain
GC: Gastric cancer
IRES: Internal ribosome entry site
NES: Nuclear export sequence
ORF: Open reading frame
PARP1: Poly ADP-ribose polymerase 1
PRDX2: Peroxiredoxin 2
RIP: RNA immunoprecipitation
RNA-FISH: RNA fluorescence in situ hybridization
TRAP: Tagged RNA affinity purification
