# Supplementary figures and images for: CircDIDO1 inhibits gastric cancer progression by encoding a novel DIDO1-529aa protein and regulating PRDX2 protein stability
Source: Mol Cancer. 2021 Aug 12;20:101. doi: 10.1186/s12943-021-01390-y (PMC8359101; doi:10.1186/s12943-021-01390-y)

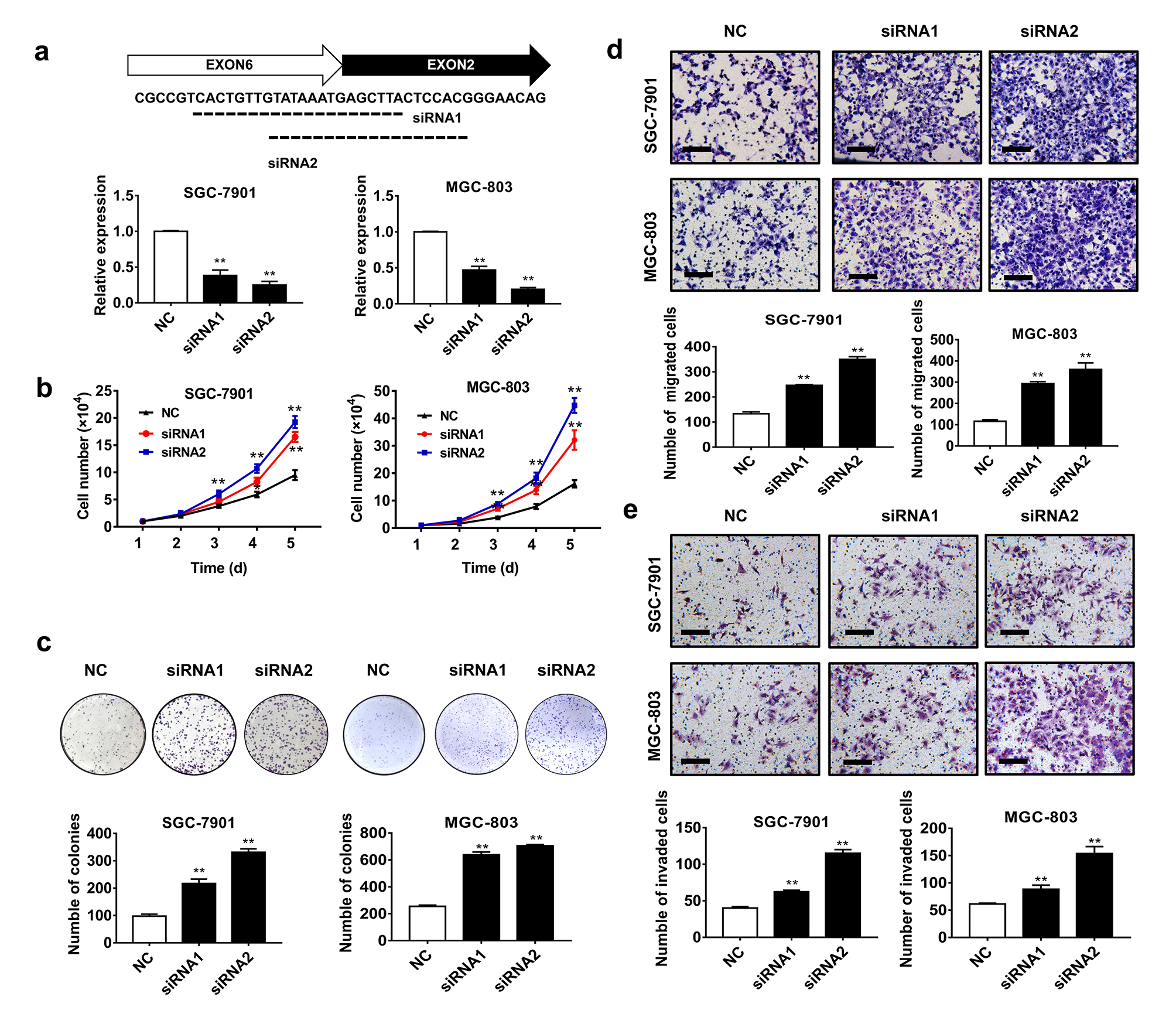

Supplement: Supplementary file 1 — Additional file 1: Figure S1. CircDIDO1 knockdown promotes GC cell proliferation, migration, and invasion in vitro. a) Efficiency of gene knockdown in GC cells by siRNAs was confirmed by qRT-PCR. b) Cell growth curve, c) colony formation, d) transwell migration, and e) matrigel invasion assays for control and circDIDO1 knockdown GC cells. [file 12943_2021_1390_MOESM1_ESM.tif]

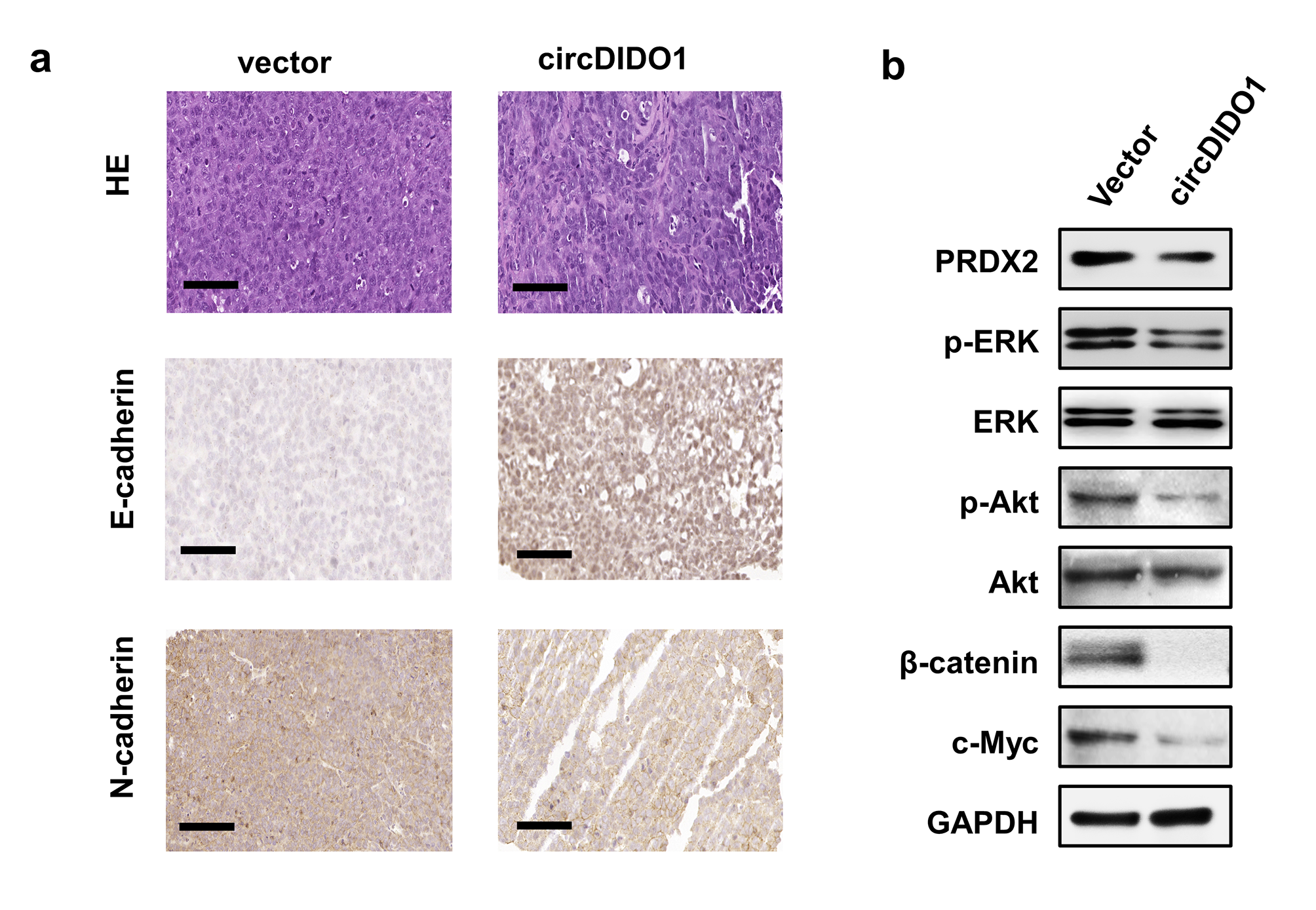

Supplement: Supplementary file 2 — Additional file 2: Figure S2. a) HE staining and immunohistochemical staining of EMT markers in liver metastasis tumors. Scale bars = 100 μm. b) The expression of proteins associated with PRDX2 downstream signaling pathways in the tumor tissues of control and circDIDO1 groups was detected by Western blot. [file 12943_2021_1390_MOESM2_ESM.tif]

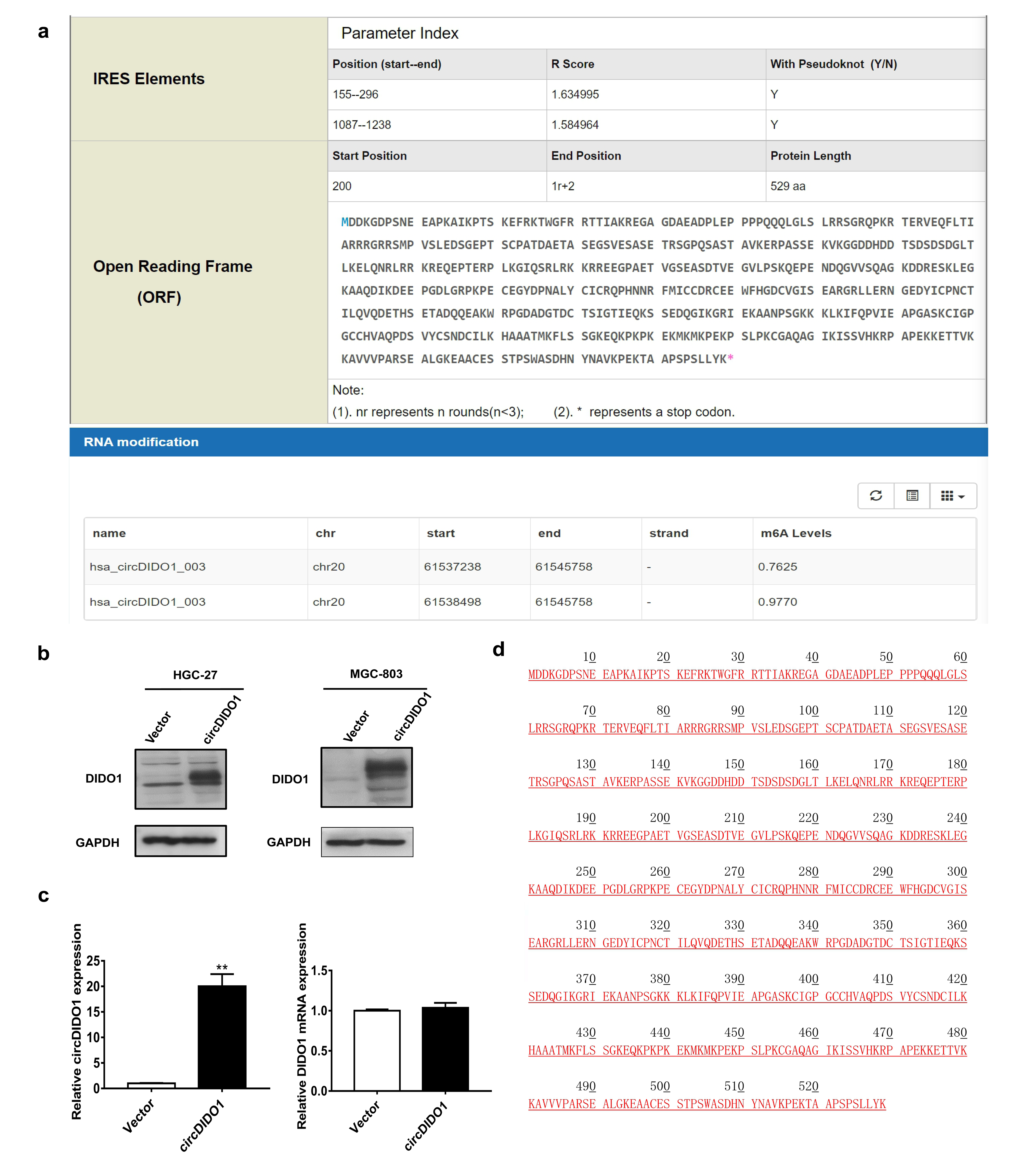

Supplement: Supplementary file 3 — Additional file 3: Figure S3. Identification of circDIDO1-encoded 529aa protein. a) Prediction of the protein-encoding ability of circDIDO1 by circRNADb software. b) Western blot assays for predicted DIDO1-509aa protein expression in GC cells by DIDO1 antibody. c)qRT-PCR analysis of circDIDO1 and DIDO1 expression in control and circDIDO1 overexpressing GC cells. d) Protein sequencing result of DIDO1-529 aa. [file 12943_2021_1390_MOESM3_ESM.tif]

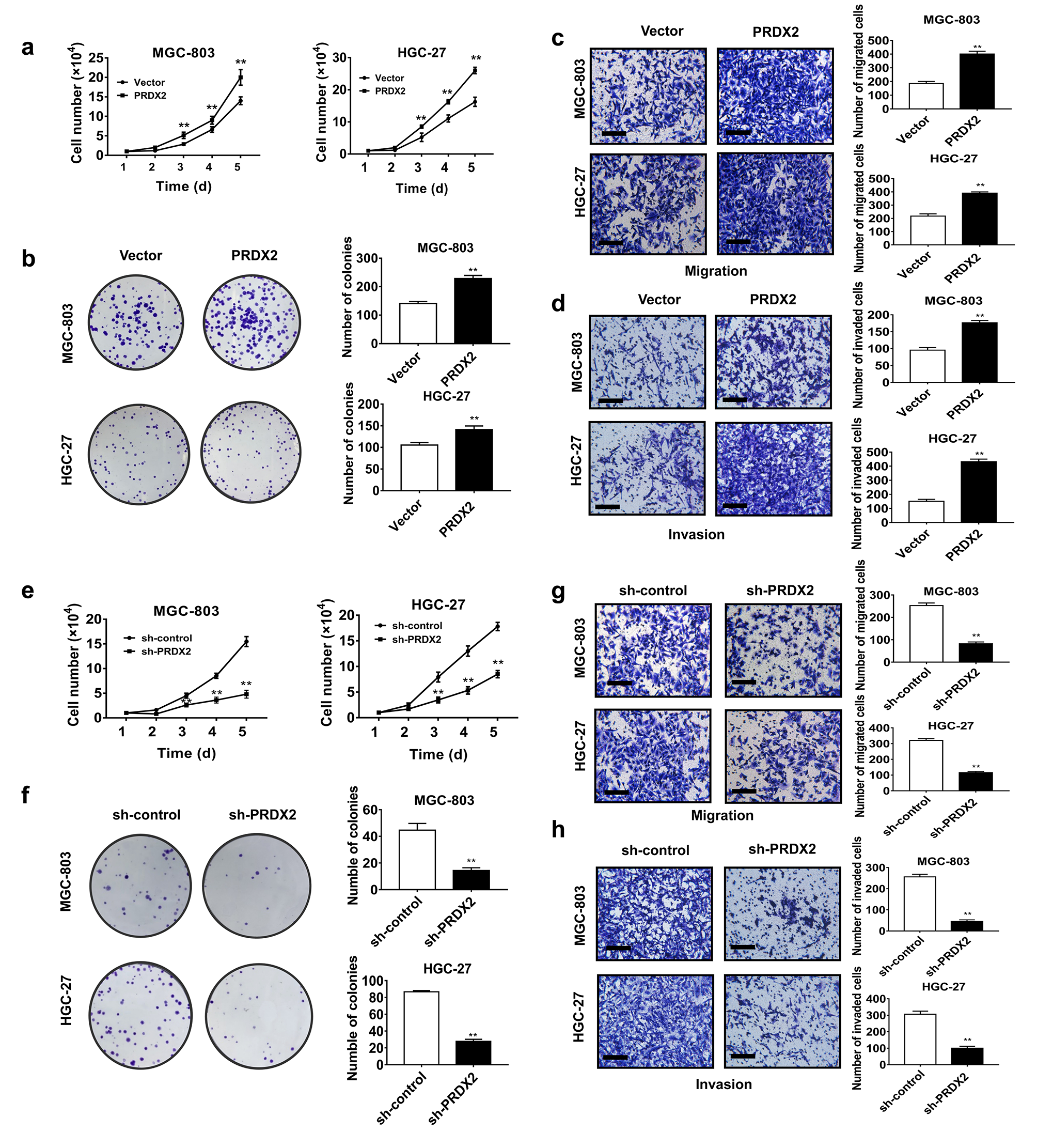

Supplement: Supplementary file 4 — Additional file 4: Figure S4. PRDX2 overexpression promotes while knockdown inhibits GC cell proliferation, migration, and invasion in vitro. a) Cell growth curve, b) colonyformation, c) transwell migration, and d) matrigel invasion assays for control and PRDX2 overexpressing GC cells. e) Cell growth curve, f) colony formation, g) transwell migration, and h) matrigel invasion assays for control and PRDX2 knockdown GC cells. [file 12943_2021_1390_MOESM4_ESM.tif]

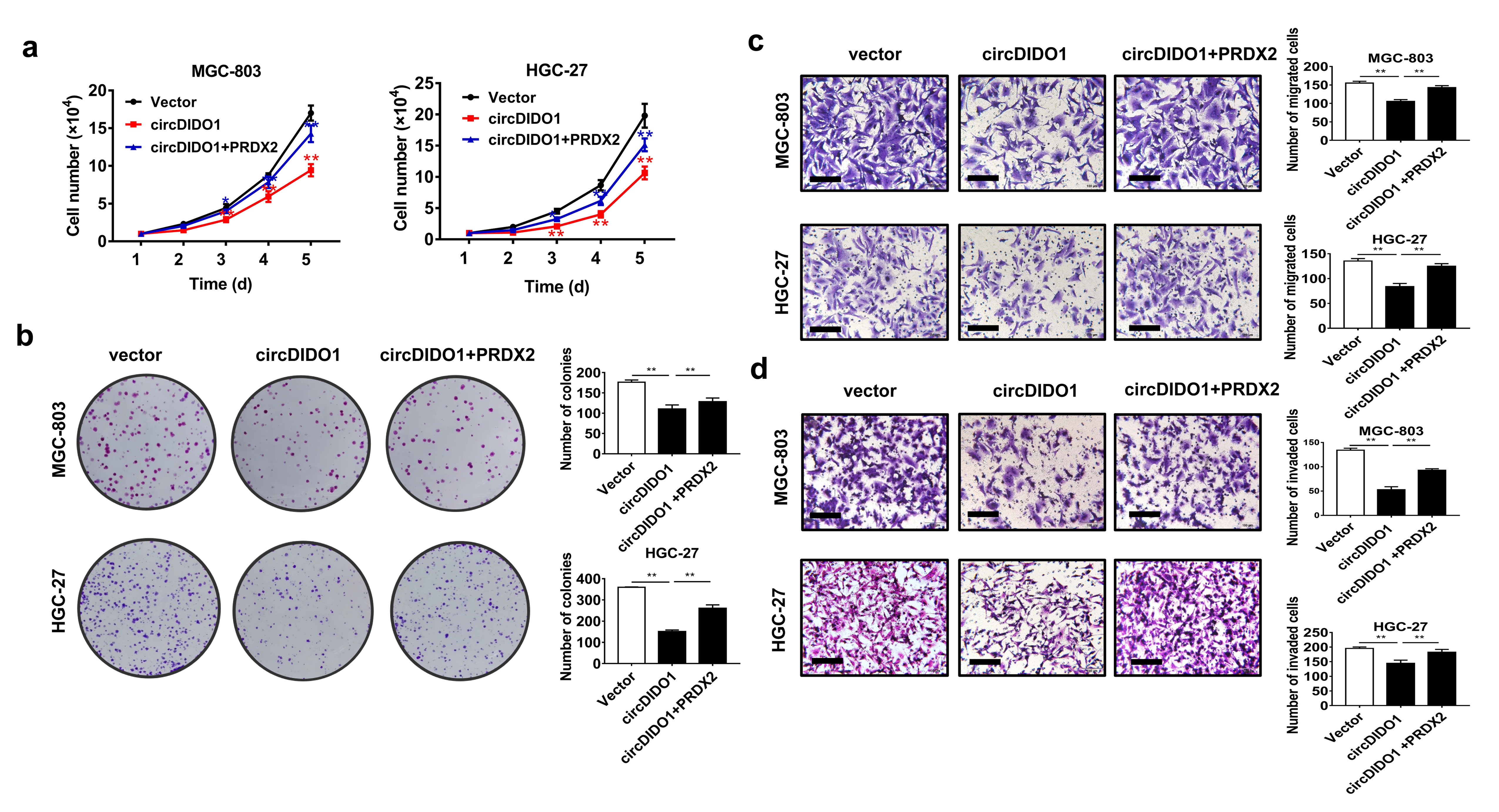

Supplement: Supplementary file 5 — Additional file 5: Figure S5. PRDX2 partially rescues the inhibition of cell proliferation, migration, and invasion in circDIDO1 overexpressing GC cells. a) Cell growth curve, b)colony formation, c) transwell migration, and d) matrigel invasion assays for circDIDO1 overexpressing GC cells co-transfected with or without PRDX2. [file 12943_2021_1390_MOESM5_ESM.tif]

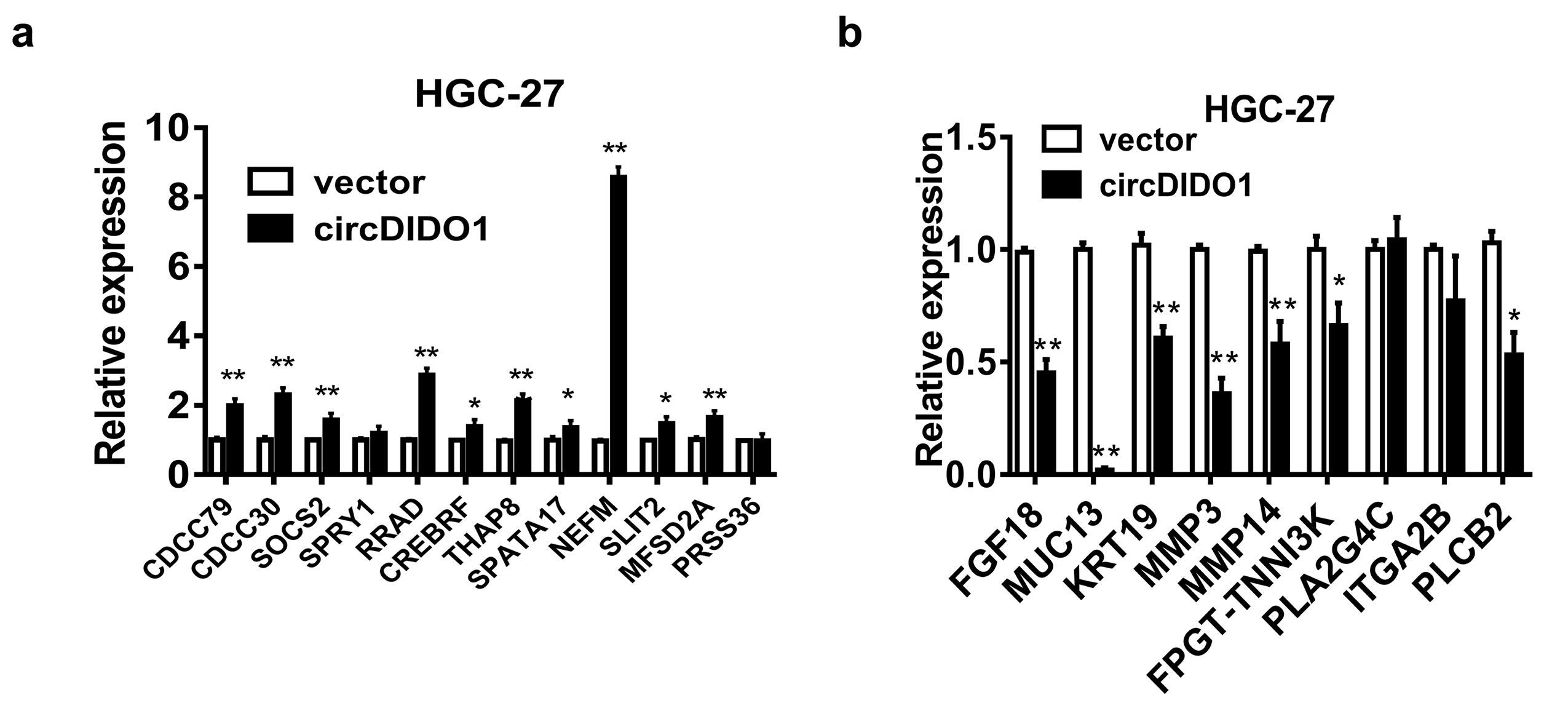

Supplement: Supplementary file 6 — Additional file 6: Figure S6. QRT-PCR analyses of differentially expressed genes in control and circDIDO1 overexpressing GC cells. [file 12943_2021_1390_MOESM6_ESM.tif]

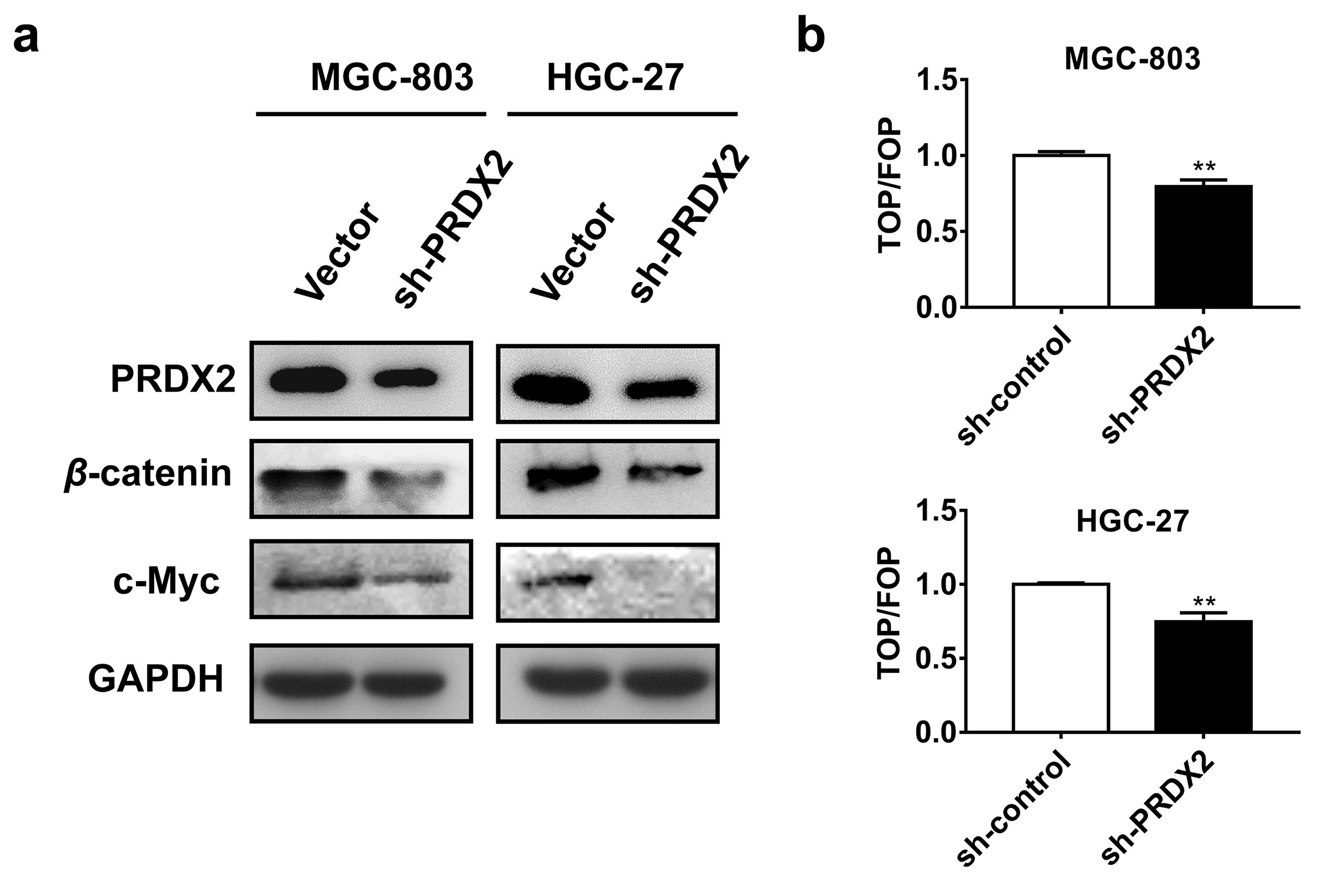

Supplement: Supplementary file 7 — Additional file 7: Figure S7. PRDX2 knockdown inhibits β-catenin expression and activity in GC cells. a) Western blot assays for protein levels of β-catenin and its downstream targets in control and PRDX2 knockdown GC cells. b) TOP/FOP flash luciferase reporter assays for β-catenin activity in control and PRDX2 knockdown GC cells. [file 12943_2021_1390_MOESM7_ESM.tif]
