# Supplementary material for: CircDIDO1 inhibits gastric cancer progression by encoding a novel DIDO1-529aa protein and regulating PRDX2 protein stability
Source: Mol Cancer. 2021 Aug 12;20:101. doi: 10.1186/s12943-021-01390-y (PMC8359101; doi:10.1186/s12943-021-01390-y)
Supplement: Supplementary file 8 — Additional file 8: Table S1. The relationship of circDIDO1 expression levels (ΔCt) in GC tissues with clinicopathological parameters. [file 12943_2021_1390_MOESM8_ESM.docx]

**Table S1.** The relationship of circDIDO1 expression levels (ΔCt) in GC tissues with clinicopathological parameters

| Characteristics | Number (%) | Mean ± SD | *p* value |
| --- | --- | --- | --- |
| Age (year) |  |  |  |
| ≥60 | 76 (74.5) | 6.05±0.53 | 0.746 |
| <60 | 26 (25.5) | 5.67±1.14 |  |
| Gender |  |  |  |
| Male | 71 (69.6) | 5.84±0.60 | 0.680 |
| Female | 31 (30.4) | 6.28±0.74 |  |
| Diameter (cm) |  |  |  |
| ≥5 | 43 (42.2) | 7.32±0.72 | 0.017* |
| <5 | 59 (57.8) | 5.00±0.62 |  |
| Differentiation |  |  |  |
| Moderate and Well | 46 (45.1) | 5.35±0.69 | 0.256 |
| Poor | 56 (54.9) | 6.45 ±0.66 |  |
| Invasion |  |  |  |
| T1 and T2 | 4 (3.9) | 2.31±2.68 | 0.106 |
| T3 and T4 | 98 (96.1) | 6.00±0.48 |  |
| Lymphatic metastasis |  |  |  |
| N0 | 27 (26.5) | 6.14±0.63 | 0.887 |
| N1 | 16 (15.7) | 5.45±1.56 |  |
| N2 | 24 (23.5) | 6.53±0.98 |  |
| N3 | 35 (34.3) | 5.66±0.92 |  |
| Distal metastasis |  |  |  |
| M0 | 95 (0.93) | 5.93±0.46 | 0.028 * |
| M1 | 7 (6.9) | 7.45±0.46 |  |
| TNM stage |  |  |  |
| I | 3 (2.9) | 4.89±1.04 | 0.801 |
| II | 29 (28.4) | 6.08±0.77 |  |
| III | 63 (61.8) | 5.91±0.63 |  |
| IV | 7 (6.9) | 7.45±0.46 |  |
| Venous invasion |  |  |  |
| Absent | 86 (84.3) | 5.89±0.52 | 0.700 |
| Present | 16 (15.7) | 6.47±1.29 |  |
| Perineural invasion |  |  |  |
| Absent | 82 (80.4) | 6.41±0.50 | 0.032 * |
| Present | 20 (19.6) | 3.64±1.32 |  |
| CA19-9 |  |  |  |
| Negative | 76 (74.5) | 6.28±0.57 | 0.216 |
| Positive | 26 (25.5) | 4.57±1.44 |  |
