# Supplementary material for: CircDIDO1 inhibits gastric cancer progression by encoding a novel DIDO1-529aa protein and regulating PRDX2 protein stability
Source: Mol Cancer. 2021 Aug 12;20:101. doi: 10.1186/s12943-021-01390-y (PMC8359101; doi:10.1186/s12943-021-01390-y)
Supplement: Supplementary file 9 — Additional file 9: Table S2. The proteins enriched in circDIDO1-MS2 group. [file 12943_2021_1390_MOESM9_ESM.docx]

**Table S2.** The proteins enriched in circDIDO1-MS2 group

| **Accession** | **Gene Symbol** | **PValue** | **FC(circDIDO1-MS2/MS2)** |
| --- | --- | --- | --- |
| NP_005546 | *KRT6B* | 0.0456 | 7.657 |
| NP_008937 | *NUDT21* | 0.0384 | 2.950 |
| NP_037364 | *PDCD6* | 0.0158 | 2.913 |
| NP_003625 | *NIPSNAP1* | 0.0371 | 2.545 |
| NP_937818 | *NME1* | 0.0301 | 2.485 |
| NP_000217 | *KRT9* | 0.0122 | 2.418 |
| XP_005246300 | *LANCL1* | 0.0461 | 2.401 |
| NP_005820 | *AP3S2* | 0.0002 | 2.389 |
| NP_001275 | *AP3S1* | 0.0028 | 2.287 |
| NP_003085 | *SNRPE* | 0.0329 | 2.183 |
| NP_006112 | *KRT1* | 0.0165 | 2.163 |
| NP_000415 | *KRT5* | 0.0340 | 2.053 |
| NP_115742 | *METTL26* | 0.0457 | 2.051 |
| NP_005498 | *CFL1* | 0.0331 | 2.035 |
| NP_066953 | *PPIA* | 0.0208 | 1.951 |
| NP_872590 | *PCNA* | 0.0376 | 1.922 |
| NP_001474 | *NIPSNAP2* | 0.0296 | 1.919 |
| NP_001307192 | *AP3M1* | 0.0408 | 1.917 |
| NP_061820 | *CYCS* | 0.0403 | 1.735 |
| NP_001027555 | *PQBP1* | 0.0067 | 1.698 |
| NP_001333790 | *TFIP11* |  | only detected in circDIDO1-MS2 group |
| NP_002956 | *S100A9* |  | only detected in circDIDO1-MS2 group |
| NP_003087 | *SNRPG* |  | only detected in circDIDO1-MS2 group |
| NP_003357 | *UQCRC2* |  | only detected in circDIDO1-MS2 group |
| NP_005611 | *S100A11* |  | only detected in circDIDO1-MS2 group |
| NP_005800 | *PRDX2* |  | only detected in circDIDO1-MS2 group |
| NP_987095 | *DDX42* |  | only detected in circDIDO1-MS2 group |
