# Supplementary material for: CircDIDO1 inhibits gastric cancer progression by encoding a novel DIDO1-529aa protein and regulating PRDX2 protein stability
Source: Mol Cancer. 2021 Aug 12;20:101. doi: 10.1186/s12943-021-01390-y (PMC8359101; doi:10.1186/s12943-021-01390-y)
Supplement: Supplementary file 10 — Additional file 10: Table S3. The potential interacting proteins enriched in PRDX2 group. [file 12943_2021_1390_MOESM10_ESM.docx]

**Table S3.** The proteins enriched in PRDX2 group

| Master Protein Accession | Entry name | Coverage[%] | Peptides | MW [kDa] |
| --- | --- | --- | --- | --- |
| P32119 | PRDX2 | 83 | 17 | 21.9 |
| Q13885 | TBB2A | 63 | 23 | 49.9 |
| P36873 | PP1G | 38 | 10 | 37 |
| P0CG39 | POTEJ | 6 | 5 | 117.3 |
| Q58FF8 | H90B2 | 17 | 8 | 44.3 |
| Q5QNW6 | H2B2F | 41 | 5 | 13.9 |
| P12235 | ADT1 | 22 | 6 | 33 |
| P12236 | ADT3 | 25 | 7 | 32.8 |
| Q13162 | PRDX4 | 11 | 4 | 30.5 |
| P13674 | P4HA1 | 8 | 3 | 61 |
| P21108 | PRPS3 | 12 | 3 | 34.8 |
| Q9UBQ0 | VPS29 | 25 | 3 | 20.5 |
| Q5T9A4 | ATD3B | 5 | 3 | 72.5 |
| A6NHQ2 | FBLL1 | 7 | 2 | 34.8 |
| P52294 | IMA5 | 8 | 3 | 60.2 |
| Q9H853 | TBA4B | 11 | 2 | 27.5 |
| Q8N5N7 | RM50 | 12 | 1 | 18.3 |
| Q9BS26 | ERP44 | 9 | 2 | 46.9 |
| P28331 | NDUS1 | 4 | 2 | 79.4 |
| Q14498 | RBM39 | 7 | 3 | 59.3 |
| P55735 | SEC13 | 11 | 3 | 35.5 |
| Q15102 | PA1B3 | 16 | 2 | 25.7 |
| P51148 | RAB5C | 11 | 2 | 23.5 |
| P98179 | RBM3 | 11 | 1 | 17.2 |
| Q6PL18 | ATAD2 | 2 | 3 | 158.5 |
| P09001 | RM03 | 9 | 2 | 38.6 |
| P02768 | ALBU | 4 | 3 | 69.3 |
| Q9UJX3 | APC7 | 3 | 1 | 66.8 |
| Q9Y4P1 | ATG4B | 4 | 1 | 44.3 |
| P61077 | UB2D3 | 29 | 2 | 16.7 |
| P69905 | HBA | 22 | 2 | 15.2 |
| Q7KZN9 | COX15 | 4 | 1 | 46 |
| O00217 | NDUS8 | 9 | 1 | 23.7 |
| Q9GZV4 | IF5A2 | 13 | 2 | 16.8 |
| O60783 | RT14 | 12 | 1 | 15.1 |
| A1L0T0 | ILVBL | 3 | 2 | 67.8 |
| O60502 | OGA | 2 | 2 | 102.8 |
| Q96LR5 | UB2E2 | 9 | 2 | 22.2 |
| P13929 | ENOB | 3 | 1 | 47 |
| P51153 | RAB13 | 11 | 2 | 22.8 |
| O75396 | SC22B | 6 | 1 | 24.6 |
| Q8N6H7 | ARFG2 | 2 | 1 | 56.7 |
| Q9Y490 | TLN1 | 1 | 2 | 269.6 |
| Q9HCJ0 | TNR6C | 1 | 2 | 175.9 |
| P19474 | RO52 | 4 | 1 | 54.1 |
| Q9NS69 | TOM22 | 8 | 1 | 15.5 |
| P41743 | KPCI | 2 | 1 | 68.2 |
| Q9HB71 | CYBP | 7 | 2 | 26.2 |
| O60518 | RNBP6 | 1 | 1 | 124.6 |
| Q86YZ3 | HORN | 2 | 1 | 282.2 |
| O43765 | SGTA | 5 | 1 | 34 |
| Q8WUD1 | RAB2B | 6 | 1 | 24.2 |
| P27824 | CALX | 4 | 2 | 67.5 |
| Q9UJZ1 | STML2 | 5 | 1 | 38.5 |
| P61201 | CSN2 | 3 | 1 | 51.6 |
| Q92688 | AN32B | 7 | 2 | 28.8 |
| Q8TB72 | PUM2 | 1 | 1 | 114.1 |
| Q9GZN8 | CT027 | 8 | 1 | 19.3 |
| P19338 | NUCL | 2 | 2 | 76.6 |
| O75390 | CISY | 2 | 1 | 51.7 |
| P57088 | TMM33 | 5 | 1 | 28 |
| Q9BVP2 | GNL3 | 2 | 1 | 62 |
| Q86UA1 | PRP39 | 2 | 1 | 78.4 |
| Q99717 | SMAD5 | 2 | 1 | 52.2 |
| Q8NE71 | ABCF1 | 2 | 1 | 95.9 |
| Q14004 | CDK13 | 2 | 1 | 164.8 |
| P35241 | RADI | 2 | 1 | 68.5 |
| P02100 | HBE | 7 | 1 | 16.2 |
| P35250 | RFC2 | 5 | 1 | 39.1 |
| Q8N766 | EMC1 | 2 | 2 | 111.7 |
| P51648 | AL3A2 | 2 | 1 | 54.8 |
| Q8WVJ2 | NUDC2 | 7 | 1 | 17.7 |
| Q5BJD5 | TM41B | 7 | 1 | 32.5 |
| Q9P2X0 | DPM3 | 11 | 1 | 10.1 |
| Q9UMS4 | PRP19 | 3 | 1 | 55.1 |
| O43324 | MCA3 | 6 | 1 | 19.8 |
| P50750 | CDK9 | 3 | 1 | 42.8 |
| Q9Y4W6 | AFG32 | 1 | 1 | 88.5 |
| Q9BQB6 | VKOR1 | 8 | 1 | 18.2 |
| P31948 | STIP1 | 2 | 1 | 62.6 |
| P50570 | DYN2 | 3 | 2 | 98 |
| P61421 | VA0D1 | 3 | 1 | 40.3 |
| Q9NQ29 | LUC7L | 3 | 1 | 43.7 |
| P08559 | ODPA | 3 | 1 | 43.3 |
| Q9H2U2 | IPYR2 | 3 | 1 | 37.9 |
| Q02978 | M2OM | 4 | 1 | 34 |
| P62891 | RL39 | 20 | 1 | 6.4 |
| Q12768 | WASC5 | 1 | 1 | 134.2 |
| Q9UKY1 | ZHX1 | 1 | 1 | 98 |
| P48556 | PSMD8 | 5 | 2 | 39.6 |
| Q13573 | SNW1 | 4 | 2 | 61.5 |
| Q9H5Q4 | TFB2M | 2 | 1 | 45.3 |
| P00568 | KAD1 | 7 | 1 | 21.6 |
| P17980 | PRS6A | 3 | 1 | 49.2 |
| O00767 | ACOD | 5 | 1 | 41.5 |
| Q99943 | PLCA | 7 | 1 | 31.7 |
| Q9NT62 | ATG3 | 3 | 1 | 35.8 |
| Q9H089 | LSG1 | 2 | 1 | 75.2 |
| Q5JPE7 | NOMO2 | 2 | 2 | 139.4 |
| O94979 | SC31A | 1 | 1 | 132.9 |
| P41214 | EIF2D | 2 | 1 | 64.7 |
| Q00341 | VIGLN | 1 | 1 | 141.4 |
| Q86Y56 | DAAF5 | 1 | 1 | 93.5 |
| Q9Y2R5 | RT17 | 7 | 1 | 14.5 |
| Q9BRU9 | UTP23 | 5 | 1 | 28.4 |
| P62877 | RBX1 | 13 | 1 | 12.3 |
| P62195 | PRS8 | 3 | 1 | 45.6 |
| Q01968 | OCRL | 1 | 1 | 104.1 |
| Q8NBI6 | XXLT1 | 2 | 1 | 43.8 |
| Q8NFF5 | FAD1 | 2 | 1 | 65.2 |
| O43617 | TPPC3 | 4 | 1 | 20.3 |
| Q9Y3T9 | NOC2L | 1 | 1 | 84.9 |
| Q96T58 | MINT | 0 | 1 | 402 |
| Q9BSU3 | NAA11 | 4 | 1 | 26 |
| P14678 | RSMB | 3 | 1 | 24.6 |
| P53597 | SUCA | 5 | 1 | 36.2 |
| Q9Y6Y0 | NS1BP | 2 | 1 | 71.7 |
| Q9Y320 | TMX2 | 4 | 1 | 34 |
| Q9HCE1 | MOV10 | 1 | 1 | 113.6 |
| Q96D46 | NMD3 | 3 | 1 | 57.6 |
| O95831 | AIFM1 | 2 | 1 | 66.9 |
| Q9Y2W1 | TR150 | 1 | 1 | 108.6 |
| Q9UN86 | G3BP2 | 2 | 1 | 54.1 |
| O43663 | PRC1 | 1 | 1 | 71.6 |
| P47897 | SYQ | 1 | 1 | 87.7 |
| Q8IYT4 | KATL2 | 2 | 1 | 61.2 |
| Q99808 | S29A1 | 2 | 1 | 50.2 |
| P36406 | TRI23 | 1 | 1 | 64 |
| O95399 | UTS2 | 6 | 1 | 14.3 |
| Q2TBE0 | C19L2 | 1 | 1 | 103.7 |
| Q9NZR2 | LRP1B | 1 | 1 | 515.2 |
| P06400 | RB | 1 | 1 | 106.1 |
| Q9C0A0 | CNTP4 | 1 | 1 | 145.2 |
| Q9Y4C2 | TCAF1 | 1 | 1 | 102.1 |
| P30085 | KCY | 9 | 1 | 22.2 |
| Q6PFW1 | VIP1 | 1 | 1 | 159.4 |
| O95793 | STAU1 | 2 | 1 | 63.1 |
| Q9ULT8 | HECD1 | 0 | 1 | 289.2 |
| O75439 | MPPB | 2 | 1 | 54.3 |
| P49207 | RL34 | 6 | 1 | 13.3 |
| Q14019 | COTL1 | 4 | 1 | 15.9 |
| Q9NPQ8 | RIC8A | 1 | 1 | 59.7 |
| Q8TEA1 | NSUN6 | 2 | 1 | 51.7 |
| Q15527 | SURF2 | 4 | 1 | 29.6 |
| Q9NZW5 | MPP6 | 1 | 1 | 61.1 |
| P21953 | ODBB | 3 | 1 | 43.1 |
| Q16762 | THTR | 2 | 1 | 33.4 |
| O75746 | CMC1 | 1 | 1 | 74.7 |
| Q05D32 | CTSL2 | 2 | 1 | 53 |
| A6NE01 | F186A | 1 | 1 | 262.6 |
| Q7Z333 | SETX | 0 | 1 | 302.7 |
| P0DP25 | CALM3 | 11 | 1 | 16.8 |
| Q86UK5 | LBN | 1 | 1 | 147.9 |
| Q8WVM8 | SCFD1 | 3 | 1 | 72.3 |
| P42858 | HD | 0 | 1 | 347.4 |
| Q9NSK0 | KLC4 | 1 | 1 | 68.6 |
| P03928 | ATP8 | 13 | 1 | 8 |
| Q02641 | CACB1 | 3 | 1 | 65.7 |
| Q8TCU4 | ALMS1 | 0 | 1 | 460.8 |
| P20700 | LMNB1 | 2 | 1 | 66.4 |
| Q2PPJ7 | RGPA2 | 1 | 1 | 210.6 |
| A6NN06 | U633A | 6 | 1 | 10.6 |
| Q9H2G2 | SLK | 1 | 1 | 142.6 |
| Q58EX2 | SDK2 | 1 | 1 | 239.2 |
| P41240 | CSK | 2 | 1 | 50.7 |
| P21802 | FGFR2 | 1 | 1 | 92 |
| Q93070 | NAR4 | 3 | 1 | 35.9 |
| Q7L2J0 | MEPCE | 1 | 1 | 74.3 |
| O95180 | CAC1H | 1 | 1 | 259 |
