# Supplementary material for: CircDIDO1 inhibits gastric cancer progression by encoding a novel DIDO1-529aa protein and regulating PRDX2 protein stability
Source: Mol Cancer. 2021 Aug 12;20:101. doi: 10.1186/s12943-021-01390-y (PMC8359101; doi:10.1186/s12943-021-01390-y)
Supplement: Supplementary file 11 — Additional file 11: Table S4. The genes dysregulated in circDIDO1 overexpression cells (mRNA-seq). [file 12943_2021_1390_MOESM11_ESM.docx]

**Table S4.** The genes dysregulated in circDIDO1 overexpression cells (mRNA-seq)

| **gene** | **gene_id** | **Fold change** | **p_value** | **Regulation** |
| --- | --- | --- | --- | --- |
| *ARHGAP33* | ENSG00000004777 | 1.758643428 | 0.02215 | up |
| *IFT88* | ENSG00000032742 | 1.755736084 | 0.0114 | up |
| *ADAMTS6* | ENSG00000049192 | 1.863850312 | 0.0049 | up |
| *LPAR2* | ENSG00000064547 | 1.880996286 | 0.01555 | up |
| *KIFAP3* | ENSG00000075945 | 1.506107764 | 0.001 | up |
| *TM9SF3* | ENSG00000077147 | 1.511015018 | 0.00325 | up |
| *ACER3* | ENSG00000078124 | 1.734826036 | 0.0025 | up |
| *PPEF1* | ENSG00000086717 | 2.539034503 | 0.0215 | up |
| *OAS1* | ENSG00000089127 | 3.261102981 | 0.00585 | up |
| *TNRC6B* | ENSG00000100354 | 1.512727367 | 0.0012 | up |
| *DIDO1* | ENSG00000101191 | 353.4837294 | 0.00005 | up |
| *POLI* | ENSG00000101751 | 1.899187046 | 0.0002 | up |
| *NALCN* | ENSG00000102452 | 3.602678718 | 0.0057 | up |
| *UGGT2* | ENSG00000102595 | 1.724483111 | 0.0013 | up |
| *UBE2W* | ENSG00000104343 | 1.633787103 | 0.01165 | up |
| *GDAP1* | ENSG00000104381 | 1.698083026 | 0.0101 | up |
| *NEFM* | ENSG00000104722 | 5.265863044 | 0.00005 | up |
| *FCGRT* | ENSG00000104870 | 3.253404036 | 0.00035 | up |
| *CHN2* | ENSG00000106069 | 2.104766221 | 0.01635 | up |
| *FSCN3* | ENSG00000106328 | 15.74148782 | 0.00305 | up |
| *PRUNE2* | ENSG00000106772 | 1.550834785 | 0.02 | up |
| *TRDMT1* | ENSG00000107614 | 1.724489088 | 0.0056 | up |
| *HOXB6* | ENSG00000108511 | 1.848892058 | 0.02125 | up |
| *GLRB* | ENSG00000109738 | 1.99130333 | 0.0061 | up |
| *NEDD9* | ENSG00000111859 | 1.544004546 | 0.00005 | up |
| *MDGA1* | ENSG00000112139 | 2.699870108 | 0.01585 | up |
| *COL4A3BP* | ENSG00000113163 | 1.527898811 | 0.011 | up |
| *ARL6* | ENSG00000113966 | 1.55923567 | 0.02455 | up |
| *ACAP2* | ENSG00000114331 | 1.553382373 | 0.0042 | up |
| *MARK1* | ENSG00000116141 | 1.687286543 | 0.0303 | up |
| *RALGPS2* | ENSG00000116191 | 2.067374854 | 0.0006 | up |
| *C1orf21* | ENSG00000116667 | 1.967220944 | 0.0476 | up |
| *SLC35A3* | ENSG00000117620 | 1.501644178 | 0.01425 | up |
| *SLC16A7* | ENSG00000118596 | 3.399046268 | 0.00005 | up |
| *DNMT3A* | ENSG00000119772 | 1.529564558 | 0.0341 | up |
| *SOCS2* | ENSG00000120833 | 1.546534481 | 0.00415 | up |
| *ITPR2* | ENSG00000123104 | 1.745358017 | 0.01465 | up |
| *CASD1* | ENSG00000127995 | 2.204436622 | 0.00155 | up |
| *APOE* | ENSG00000130203 | 2.353421938 | 0.035 | up |
| *RSPH3* | ENSG00000130363 | 1.693732453 | 0.01015 | up |
| *NPAS1* | ENSG00000130751 | 2.281053051 | 0.00565 | up |
| *GMFG* | ENSG00000130755 | inf | 0.00005 | up |
| *CCDC62* | ENSG00000130783 | 2.394293478 | 0.00115 | up |
| *LHX1* | ENSG00000132130 | 3.133033075 | 0.04785 | up |
| *GSTT2B* | ENSG00000133433 | 1.761670366 | 0.0088 | up |
| *TTLL7* | ENSG00000137941 | 2.320056597 | 0.0114 | up |
| *IFI44L* | ENSG00000137959 | 1.968482654 | 0.0101 | up |
| *APH1B* | ENSG00000138613 | 1.741571858 | 0.0013 | up |
| *HERC6* | ENSG00000138642 | 2.013715678 | 0.02915 | up |
| *PDE5A* | ENSG00000138735 | 1.92094738 | 0.0226 | up |
| *EGF* | ENSG00000138798 | 4.058003192 | 0.0007 | up |
| *NAA30* | ENSG00000139977 | 1.671088338 | 0.0013 | up |
| *ABCA8* | ENSG00000141338 | 1.762621857 | 0.0299 | up |
| *SLMO1* | ENSG00000141391 | 1.651482864 | 0.00895 | up |
| *RCN3* | ENSG00000142552 | 2.487161173 | 0.0071 | up |
| *NBPF3* | ENSG00000142794 | 1.965861931 | 0.02635 | up |
| *LYPLAL1* | ENSG00000143353 | 1.514862657 | 0.00555 | up |
| *CNIH3* | ENSG00000143786 | 3.280964536 | 0.03725 | up |
| *IL17RD* | ENSG00000144730 | 1.820528089 | 0.04145 | up |
| *SLIT2* | ENSG00000145147 | 2.483973875 | 0.0114 | up |
| *TRMT10A* | ENSG00000145331 | 1.566287445 | 0.0062 | up |
| *SLC16A2* | ENSG00000147100 | 1.569704506 | 0.00855 | up |
| *TTC12* | ENSG00000149292 | 1.590765303 | 0.04235 | up |
| *THRB* | ENSG00000151090 | 1.523922991 | 0.03405 | up |
| *FER* | ENSG00000151422 | 1.67334741 | 0.0354 | up |
| *FAM160B1* | ENSG00000151553 | 1.804965172 | 0.00605 | up |
| *BEND6* | ENSG00000151917 | 1.698949535 | 0.01525 | up |
| *TMEM56* | ENSG00000152078 | 1.513622039 | 0.03095 | up |
| *GDPD1* | ENSG00000153982 | 2.69910294 | 0.0033 | up |
| *TTC39B* | ENSG00000155158 | 2.448146681 | 0.00005 | up |
| *GNAQ* | ENSG00000156052 | 1.638579874 | 0.0198 | up |
| *ZNF66* | ENSG00000160229 | 2.420994833 | 0.02235 | up |
| *IL6R* | ENSG00000160712 | 1.555047878 | 0.0255 | up |
| *FBXO27* | ENSG00000161243 | 1.820905434 | 0.03255 | up |
| *THAP8* | ENSG00000161277 | 1.726894562 | 0.0175 | up |
| *MYSM1* | ENSG00000162601 | 1.855309661 | 0.0442 | up |
| *SPATA17* | ENSG00000162814 | 1.895741166 | 0.04 | up |
| *NBPF8* | ENSG00000162825 | 2.37375329 | 0.0174 | up |
| *KIAA1841* | ENSG00000162929 | 1.61416941 | 0.0131 | up |
| *HDAC11* | ENSG00000163517 | 1.558848799 | 0.0228 | up |
| *C3orf67* | ENSG00000163689 | 1.761990322 | 0.04945 | up |
| *SPRY1* | ENSG00000164056 | 1.86684222 | 0.03205 | up |
| *GRIK2* | ENSG00000164418 | 1.884073177 | 0.02275 | up |
| *CREBRF* | ENSG00000164463 | 1.747292333 | 0.0074 | up |
| *RAPSN* | ENSG00000165917 | 11.89464245 | 0.00025 | up |
| *TMEM130* | ENSG00000166448 | 1.700534175 | 0.0139 | up |
| *RRAD* | ENSG00000166592 | 2.937054426 | 0.04895 | up |
| *TRANK1* | ENSG00000168016 | 2.531758859 | 0.02285 | up |
| *POLR2J3* | ENSG00000168255 | 1.57699272 | 0.0355 | up |
| *MFSD2A* | ENSG00000168389 | 2.033168804 | 0.00515 | up |
| *FAM84B* | ENSG00000168672 | 2.115516591 | 0.01855 | up |
| *IL7R* | ENSG00000168685 | 1.509664533 | 0.016 | up |
| *CXCL10* | ENSG00000169245 | 2.084844814 | 0.00805 | up |
| *PDGFD* | ENSG00000170962 | 2.291369305 | 0.00365 | up |
| *EFEMP2* | ENSG00000172638 | 10.26648204 | 0.0003 | up |
| *HPSE* | ENSG00000173083 | 1.614486078 | 0.00415 | up |
| *MSRB3* | ENSG00000174099 | 1.666647496 | 0.0026 | up |
| *TLR6* | ENSG00000174130 | 1.507205359 | 0.041 | up |
| *FAM53A* | ENSG00000174137 | 2.052937918 | 0.0499 | up |
| *ANKRD36C* | ENSG00000174501 | 1.600215273 | 0.01865 | up |
| *THAP6* | ENSG00000174796 | 1.793602259 | 0.03 | up |
| *GK5* | ENSG00000175066 | 1.573902313 | 0.00435 | up |
| *PRSS36* | ENSG00000178226 | 2.31052388 | 0.0443 | up |
| *PCGF5* | ENSG00000180628 | 1.655777707 | 0.00045 | up |
| *BBS12* | ENSG00000181004 | 1.670591497 | 0.0081 | up |
| *FANCB* | ENSG00000181544 | 1.574327839 | 0.0327 | up |
| *ZNF708* | ENSG00000182141 | 1.983279489 | 0.00935 | up |
| *RAD51B* | ENSG00000182185 | 1.849881683 | 0.0207 | up |
| *SATB1* | ENSG00000182568 | 2.388922415 | 0.03055 | up |
| *SLC25A21* | ENSG00000183032 | 2.396950308 | 0.02295 | up |
| *PRR16* | ENSG00000184838 | 1.789578712 | 0.0185 | up |
| *USP18* | ENSG00000184979 | 1.541748027 | 0.00875 | up |
| *POMK* | ENSG00000185900 | 1.553035707 | 0.0482 | up |
| *PTCH1* | ENSG00000185920 | 1.546560209 | 0.03235 | up |
| *CCDC30* | ENSG00000186409 | 10.78317408 | 0.002 | up |
| *GCNT1* | ENSG00000187210 | 1.599201799 | 0.00335 | up |
| *RINL* | ENSG00000187994 | 10.82031054 | 0.00045 | up |
| *MYO6* | ENSG00000196586 | 1.619086428 | 0.0172 | up |
| *ACSL5* | ENSG00000197142 | 3.195474113 | 0.00515 | up |
| *OGDHL* | ENSG00000197444 | 2.08630488 | 0.0021 | up |
| *BCO2* | ENSG00000197580 | 4.37857068 | 0.01015 | up |
| *F8A1* | ENSG00000197932 | 2.025320181 | 0.00205 | up |
| *FAM115A* | ENSG00000198420 | 1.806723833 | 0.01265 | up |
| *F8A2* | ENSG00000198444 | 1.816644342 | 0.00655 | up |
| *C1orf228* | ENSG00000198520 | 2.106079652 | 0.02595 | up |
| *INPP5F* | ENSG00000198825 | 1.635597769 | 0.0388 | up |
| *ZNF525* | ENSG00000203326 | 1.714026007 | 0.03585 | up |
| *LYRM5* | ENSG00000205707 | 1.574821157 | 0.04585 | up |
| *C14orf132* | ENSG00000227051 | 2.043496822 | 0.01805 | up |
| *WASH4P* | ENSG00000234769 | 2.331647989 | 0.0012 | up |
| *KLHL41* | ENSG00000239474 | 2.050122334 | 0.0295 | up |
| *HBB* | ENSG00000244734 | 6.809741339 | 0.00005 | up |
| *HP* | ENSG00000257017 | 42.72711493 | 0.00005 | up |
| *AC139100.2* | ENSG00000267270 | 2.882678611 | 0.01725 | up |
| *ITGA2B* | ENSG00000005961 | -3.665550801 | 0.0017 | down |
| *RUNX3* | ENSG00000020633 | -1.518423263 | 0.0156 | down |
| *CHRDL2* | ENSG00000054938 | -inf | 0.01855 | down |
| *CDK17* | ENSG00000059758 | -1.509973259 | 0.0242 | down |
| *PPP2R5B* | ENSG00000068971 | -1.692045073 | 0.00435 | down |
| *SMOX* | ENSG00000088826 | -1.693127948 | 0.00005 | down |
| *SLC30A4* | ENSG00000104154 | -3.689793301 | 0.0357 | down |
| *LIN7B* | ENSG00000104863 | -1.712574798 | 0.0478 | down |
| *PLA2G4C* | ENSG00000105499 | -2.083241365 | 0.0112 | down |
| *KCNIP3* | ENSG00000115041 | -1.631141568 | 0.00685 | down |
| *PCSK4* | ENSG00000115257 | -3.764374077 | 0.0011 | down |
| *NR4A3* | ENSG00000119508 | -2.006317596 | 0.00005 | down |
| *SLC10A7* | ENSG00000120519 | -1.758994534 | 0.01005 | down |
| *NR4A1* | ENSG00000123358 | -1.655027283 | 0.00015 | down |
| *PI3* | ENSG00000124102 | -inf | 0.00005 | down |
| *RTN2* | ENSG00000125744 | -1.558053745 | 0.0059 | down |
| *ZSCAN5A* | ENSG00000131848 | -1.584385057 | 0.0063 | down |
| *SERPINF1* | ENSG00000132386 | -2.11148793 | 0.0268 | down |
| *PLCB2* | ENSG00000137841 | -2.009853018 | 0.00625 | down |
| *STAC2* | ENSG00000141750 | -1.558831511 | 0.0005 | down |
| *SIK1* | ENSG00000142178 | -1.503491801 | 0.00005 | down |
| *HNRNPLL* | ENSG00000143889 | -1.512529206 | 0.00005 | down |
| *FAM86B2* | ENSG00000145002 | -8.936005043 | 0.00905 | down |
| *FSIP1* | ENSG00000150667 | -8.973681626 | 0.00195 | down |
| *WDR78* | ENSG00000152763 | -1.937675521 | 0.0297 | down |
| *NR4A2* | ENSG00000153234 | -1.699162698 | 0.0015 | down |
| *FGF18* | ENSG00000156427 | -1.833788176 | 0.0004 | down |
| *DMTN* | ENSG00000158856 | -1.667903707 | 0.03335 | down |
| *PKDCC* | ENSG00000162878 | -1.789649419 | 0.00005 | down |
| *MAMDC2* | ENSG00000165072 | -1.88315402 | 0.03105 | down |
| *ANXA8* | ENSG00000165390 | -1.62479308 | 0.03775 | down |
| *KRT19* | ENSG00000171345 | -2.11460764 | 0.0447 | down |
| *MUC13* | ENSG00000173702 | -1.656559474 | 0.0111 | down |
| *GTF2IRD2B* | ENSG00000174428 | -2.005886534 | 0.00255 | down |
| *TP53I11* | ENSG00000175274 | -1.587685322 | 0.037 | down |
| *ZNF613* | ENSG00000176024 | -2.106634458 | 0.0494 | down |
| *RPRM* | ENSG00000177519 | -4.155638041 | 0.00005 | down |
| *RNF182* | ENSG00000180537 | -2.367935854 | 0.02935 | down |
| *B4GALNT4* | ENSG00000182272 | -1.790395109 | 0.02565 | down |
| *RWDD4* | ENSG00000182552 | -1.670473389 | 0.00045 | down |
| *HIST1H2AK* | ENSG00000184348 | -2.208780414 | 0.0296 | down |
| *DUSP8* | ENSG00000184545 | -1.76171921 | 0.0137 | down |
| *F8A3* | ENSG00000185990 | -6.738885438 | 0.00005 | down |
| *SPIN2B* | ENSG00000186787 | -1.696065634 | 0.001 | down |
| *KRT14* | ENSG00000186847 | -inf | 0.00005 | down |
| *PPARA* | ENSG00000186951 | -1.870931839 | 0.0208 | down |
| *AGAP4* | ENSG00000188234 | -2.002663457 | 0.015 | down |
| *RASGEF1A* | ENSG00000198915 | -1.552915145 | 0.01225 | down |
| *MTRNR2L10* | ENSG00000256045 | -2.497786211 | 0.03735 | down |
| *ZNF432* | ENSG00000256087 | -1.706492413 | 0.02835 | down |
| *FPGT-TNNI3K* | ENSG00000259030 | -223.0881388 | 0.0027 | down |
| *UBBP4* | ENSG00000263563 | -1.827464018 | 0.04585 | down |
