# Supplementary material for: CircDIDO1 inhibits gastric cancer progression by encoding a novel DIDO1-529aa protein and regulating PRDX2 protein stability
Source: Mol Cancer. 2021 Aug 12;20:101. doi: 10.1186/s12943-021-01390-y (PMC8359101; doi:10.1186/s12943-021-01390-y)
Supplement: Supplementary file 12 — Additional file 12: Table S5. Primer sequences for qRT-PCR. [file 12943_2021_1390_MOESM12_ESM.docx]

**Table S5.** Primer sequences for qRT-PCR

| Name | Primer | Sequence(5'-3') |
| --- | --- | --- |
| circDIDO1 | Forward primer | TGGGCGAGCGATCACAATTAC |
|  | Reverse primer | CAGACGAAACCTCTGGGTCC |
| DIDO1 mRNA | Forward primer | AAGGAAGACAGGAGGTCCGA |
|  | Reverse primer | TGGTGCCCTTGAAACCTGAG |
| GAPDH | Forward primer | GGATTTGGTCGTATTGGG |
|  | Reverse primer | GGAAGATGGTGATGGGATT |
| PRDX2 | Forward primer | GAAGCTGTCGGACTACAAAGG |
|  | Reverse primer | TCGGTGGGGCACACAAAAG |
