# Supplementary material for: CircDIDO1 inhibits gastric cancer progression by encoding a novel DIDO1-529aa protein and regulating PRDX2 protein stability
Source: Mol Cancer. 2021 Aug 12;20:101. doi: 10.1186/s12943-021-01390-y (PMC8359101; doi:10.1186/s12943-021-01390-y)
Supplement: Supplementary file 13 — Additional file 13: Table S6. The sequences of oligonucleotides. [file 12943_2021_1390_MOESM13_ESM.docx]

**Table S6.** Oligonucleotides

| Name | Target Seq |
| --- | --- |
| NC | TTCTCCGAACGTGTCACGT |
| circDIDO1 siRNA1 | CACTGTTGTATAAATGAGC |
| circDIDO1 siRNA2 | GTATAAATGAGCTTACTCC |
| sh-control | TTCTCCGAACGTGTCACGT |
| sh-PRDX2 | CCTCTTTATCATCGATGGCAA |
| circDIDO1 FISH probe | GTTCCCGTGGAGTAAGCTCATTTATACAACAGTGACGGCG |
