# Supplementary material for: CircDIDO1 inhibits gastric cancer progression by encoding a novel DIDO1-529aa protein and regulating PRDX2 protein stability
Source: Mol Cancer. 2021 Aug 12;20:101. doi: 10.1186/s12943-021-01390-y (PMC8359101; doi:10.1186/s12943-021-01390-y)
Supplement: Supplementary file 14 — Additional file 14: Table S7. The information of antibodies used in this study. [file 12943_2021_1390_MOESM14_ESM.docx]

**Table S7.** Antibodies used in this study

| Antigens | Manufacturers | Applications |
| --- | --- | --- |
| RAPR1 | #9532, Cell Signaling Technology, Beverly, MA, USA | 1:1000 for WB |
|  |  | 1:100 for IFA |
| Flag | 66008-3-Ig, Proteintech, Wuhan, Hubei, China | 1:1000 for WB |
|  |  | 1:100 for IFA |
| PRDX2 | 10545-2-AP, Proteintech, Wuhan, Hubei, China | 1:2000 for WB |
| E-cadherin | #3195, Cell Signaling Technology, Beverly, MA, USA | 1:100 for IHC |
| N-cadherin | #13116, Cell Signaling Technology, Beverly, MA, USA | 1:100 for IHC |
| DIDO1 | 10183-1-AP, Proteintech, Wuhan, Hubei, China | 1:1000 for WB |
| cleaved PARP1 | #5625, Cell Signaling Technology, Beverly, MA, USA | 1:1000 for WB |
| HA | AH158, Beyotime Biotechnology, Shanghai, China | 1:1000 for WB |
| RBX1 | 66716-1-Ig, Proteintech, Wuhan, Hubei, China | 1:2000 for WB |
| Ub | #3936, Cell Signaling Technology, Beverly, MA, USA | 1:1000 for WB |
| cleaved caspase 3 | #9661, Cell Signaling Technology, Beverly, MA, USA | 1:1000 for WB |
| caspase 3 | #14220, Cell Signaling Technology, Beverly, MA, USA | 1:1000 for WB |
| GST | AF0174, Beyotime Biotechnology, Shanghai, China | 1:1000 for WB |
| ERK | #4695, Cell Signaling Technology, Beverly, MA, USA | 1:1000 for WB |
| p-ERK | #4376, Cell Signaling Technology, Beverly, MA, USA | 1:1000 for WB |
| Akt | #4691, Cell Signaling Technology, Beverly, MA, USA | 1:1000 for WB |
| p-Akt | #4060, Cell Signaling Technology, Beverly, MA, USA | 1:1000 for WB |
| *β*-catenin | #8480, Cell Signaling Technology, Beverly, MA, USA | 1:1000 for WB |
| c-Myc | #18583, Cell Signaling Technology, Beverly, MA, USA | 1:1000 for WB |
| γH2AX | #9718, Cell Signaling Technology, Beverly, MA, USA | 1:100 for IFA |
| Ub | Ab7780, Abcam, Cambridge, MA, USA | 1:1000 for WB |
| GAPDH | #5174, Cell Signaling Technology, Beverly, MA, USA | 1:2000 for WB |
| HRP-linked anti-rabbit IgG | #7074, Cell Signaling Technology, Beverly, MA, USA | 1:5000 for WB |
| HRP-linked anti-mouse IgG | #7076, Cell Signaling Technology, Beverly, MA, USA | 1:5000 for WB |
