# Supplementary material for: CircDIDO1 inhibits gastric cancer progression by encoding a novel DIDO1-529aa protein and regulating PRDX2 protein stability
Source: Mol Cancer. 2021 Aug 12;20:101. doi: 10.1186/s12943-021-01390-y (PMC8359101; doi:10.1186/s12943-021-01390-y)
Supplement: Supplementary file 15 — Additional file 15: Supplementary Methods. [file 12943_2021_1390_MOESM15_ESM.docx]

**Supplementary Methods**

**RNA fluorescence in situ hybridization (RNA-FISH)**

Cells seeded on the slides were fixed with 4% paraformaldehyde. Hybridization was performed with a specific probe for circDIDO1 junction site by using Fluorescent In Situ Hybridization Kit (GenePharma, Shanghai, China). Cell nuclei were counterstained with 4’,6-diamidino-2-phenylindole (DAPI).

**RNA extraction and quantitative real time PCR**

Total RNA was isolated from tissue samples and cells by using Trizol reagent (Invitrogen) according to the manufacturer’s procedures. The reverse transcription (RT) for mRNA and circRNA was carried out by using the HiScript 1st Strand cDNA Synthesis Kit (Vazyme, Nanjing, China). Quantitative real time polymerase chain reaction was conducted with UltraSYBR Mixture (Cwbio, Beijing, China) on a real time PCR Detection System (CFX96, Bio-Rad, Hercules, CA, USA). The target genes were normalized to GAPDH to obtain the relative expression level. The sequences of primers were provided in Table S5 (Supporting Information).

**Gene overexpression and silencing**

Cells were seeded in 6-well plates at a density of 2×10^5^ per well and cultured in 37 °C incubator overnight. The over-expressing plasmid and siRNA (Hanbio, Shanghai, China) were transfected into the cells by using LipoFiter transfection reagent (Hanbio) in serum-free medium. Cells were changed to complete medium at 6 h after transfection and cultured for another 30 h. The target sequences of siRNAs and shRNAs were provided in Table S6 (Supporting Information).

**Western blot**

Cells were lysed with RIPA buffer (Beyotime, Shanghai, China) containing protease inhibitors (Roche, CA, USA). Equal amounts of proteins were separated by SDS-polyacrylamide gel electrophoresis (SDS-PAGE) on a 12% polyacrylamide gel. The proteins were transferred electrophoretically onto 0.22 μm PVDF membranes (Millipore), blocked in 5% non-fat milk, and then incubated with primary antibodies. After incubation with HRP-linked secondary antibody, the protein bands were visualized by using chemiluminescence (Millipore, Shanghai, China). GAPDH was used as the loading control.

**Immunohistochemistry**

For immunohistochemical analyses, 4% paraformaldehyde fixed tissues were embedded in paraffin and cut into 4 μm-thick sections. The sections were incubated with primary monoclonal antibody against Ki-67 (Cell Signaling Technology) followed by incubation with the secondary antibody for 30 min at room temperature. After incubation with 3, 3’-Diaminobenzidine (3, 3’-DAB, Maxim, Fuzhou, China) for 5 min, the sections were counterstained with hematoxylin for 30 s. Finally, the sections were photographed under a TE2000 microscope (Nikon, Tokyo, Japan).

**Immunofluorescence**

Cells seeded on the slides were fixed with 4% paraformaldehyde, permeabilized with 0.5% Triton X-100, and blocked in 5% BSA. The target proteins were then incubated with primary antibodies at 4°C overnight. After incubation with Alexa Fluor 555 donkey anti-rabbit IgG or FITC goat anti-mouse IgG and counterstaining with DAPI, the fluorescent signals were visualized under confocal microscope.

**LC-MS/MS**

DIDO1-529aa protein full sequence analysis was carried out using rLys-C, rLys-C & Asp-N, rLys-C & Glu-C, Chymotrypsin&Glu-C and Pepsin&Glu-C protease digestion methods respectively, and finally the full protein sequence analysis was performed. Other protein mass spectrometry used trypsin for protein digestion. Before analysis, the peptides were reconstituted in 10 μL of 0.1% formic acid. LC-MS/MS was performed on an Q Exactive™ Hybrid Quadrupole-Orbitrap™ Mass Spectrometer (Thermo Scientific) coupled with Ultimate 3000 System. For each sample，5 μL of volume was loaded onto C18 PepMap100 trapcolumn (300 μm×5 mm) and eluted on a Thermo Acclaim PepMap RPLC analytical column (150 μm×15 cm). .A procedure of 60 min gradient for each single-shot analysis was performed as followed: 6-9% B in 5 min, 9-14% B in 15 min, 14-30% B in 30 min, 30-40% B in 8 min, 40-95% B in 2 min (A=0.1% formic acid in water, B=0.1% formic acid in 90% acetonitrile). The flow rate was 0.6 μL/min. Data-dependent mode was operated for the mass spectrometer, with a full MS scan (300-1400 m/z) and 3 s cycle time was set. The MS spectra were acquired at a resolution of 70,000 with an automatic gain control (AGC) target value of 3×106 ions or a maximum integration time of 40 ms. High energy collision dissociation (HCD) with the energy set at 27 NCE was used to perform peptide fragmentation. The MS/MS spectra were acquired in the top 15 or 20 most intense precursors at a resolution of 17,500 with an AGC target value of 1×105 ions or a maximum integration time of 60 ms. The raw MS files were analyzed and searched against uniprot-Homo sapiens database based on the species of the samples using Byonic. The mass tolerance was set to 20 ppm and 0.02 Da for the precursor and the fragment ion respectively, with up to three missed cleavages allowed. Carbamidomethyl (+57.021 Da) was used as a fixed modification and oxidation (M) was used as a variable modification. The results of protein identification were filtered with the criteria of mass tolerance less than 10 ppm for peptides and false positive rate less than 1% at the protein level. Only high confident identified peptides were chosen for downstream protein identification analysis.
